# Orchestrated neuronal migration and cortical folding: A computational and experimental study

**DOI:** 10.1371/journal.pcbi.1010190

**Published:** 2022-06-16

**Authors:** Shuolun Wang, Kengo Saito, Hiroshi Kawasaki, Maria A. Holland

**Affiliations:** 1 Department of Aerospace and Mechanical Engineering, University of Notre Dame, Notre Dame, Indiana, United States of America; 2 Department of Medical Neuroscience, Graduate School of Medical Sciences, Kanazawa University, Ishikawa, Japan; 3 Bioengineering Graduate Program, University of Notre Dame, Notre Dame, Indiana, United States of America; Kings College London, UNITED KINGDOM

## Abstract

Brain development involves precisely orchestrated genetic, biochemical, and mechanical events. At the cellular level, neuronal proliferation in the innermost zone of the brain followed by migration towards the outermost layer results in a rapid increase in brain surface area, outpacing the volumetric growth of the brain, and forming the highly folded cortex. This work aims to provide mechanistic insights into the process of brain development and cortical folding using a biomechanical model that couples cell division and migration with volumetric growth. Unlike phenomenological growth models, our model tracks the spatio-temporal development of cohorts of neurons born at different times, with each cohort modeled separately as an advection-diffusion process and the total cell density determining the extent of volume growth. We numerically implement our model in Abaqus/Standard (2020) by writing user-defined element (UEL) subroutines. For model calibration, we apply *in utero* electroporation (IUE) to ferret brains to visualize and track cohorts of neurons born at different stages of embryonic development. Our calibrated simulations of cortical folding align qualitatively with the ferret experiments. We have made our experimental data and finite-element implementation available online to offer other researchers a modeling platform for future study of neurological disorders associated with atypical neurodevelopment and cortical malformations.

## Author summary

Brain development and cortical folding is a highly dynamic process that results from the interaction between gene expression, cellular mechanisms, and mechanical forces. Here, we expand on existing mathematical models of brain development and cortical folding to capture the behavior of multiple different subpopulations of neurons. By calibrating our biomechanical model to our novel experiments on ferrets, we can track the distribution of neurons over time and observe how the brain grows and develops its characteristic folds. Our calibrated model captures interactions between cell behavior and tissue deformation and offers more detailed information about the orchestrated migration of neuronal subpopulations. This work offers new mechanistic insights into brain development and opens the door to future investigations of atypical brain development caused by disrupted neuronal activities, particularly those alterations associated with injury, exposure, or treatment at a specific location or time during development. Finally, our experimental data and numerical implementations are provided as a resource online for the use of other researchers.

## 1 Introduction

Cortical folding—a process that turns smooth fetal brains into convoluted adult brains—maximizes the cortical area while minimizing the length of axonal connections for a given brain volume. It is a hallmark of advanced brain function and intelligence [1, 2], offering gyrencephalic mammals superior information processing capabilities than other species with smooth or lissencephalic brains [3]. Atypical neurodevelopment and cortical malformation are associated with neurological disorders such as autism spectrum disorder [4], schizophrenia [5], and epilepsy [6]. Hence, a deeper understanding of neurodevelopment is of great interest and could prove to be essential for increased understanding, improved diagnostics, and effective treatments of developmental disorders.

**Fig 1:**
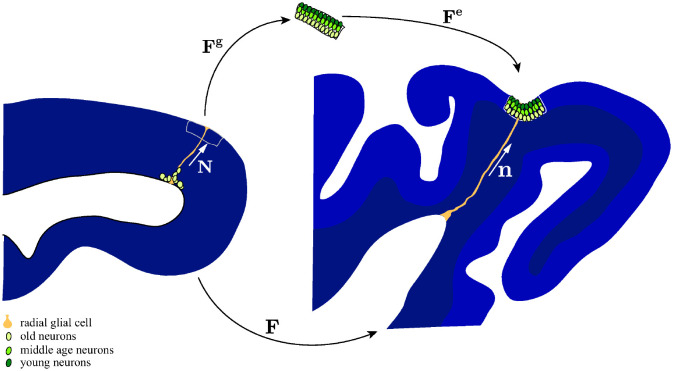
Schematic of neurodevelopment as represented in the proposed model. Intermediate progenitor cells (light green) proliferate in the subventricular zone and mature into neurons before migrating outwards along radial glial fibers (orange) to arrive at the cortical plate. There, they arrange themselves into layers, with younger neurons (dark green) bypassing existing layers to reach the pial surface (top). The radial and tangential expansion of the cortical plate results in cortical folding (right). In the proposed model, the total deformation throughout development is described by the deformation gradient **F**, which can be decomposed into components that describe the local growth as a result of cellular proliferation and migration (**F**^g^) and the passive physical deformation that accompanies cortical folding (**F**^e^). The orientation of radial glial cells in undeformed and deformed states are denoted as unit vectors **N** and **n**, respectively. For detailed explanations of these variables, please refer to Section 4.4.

**Fig 2:**
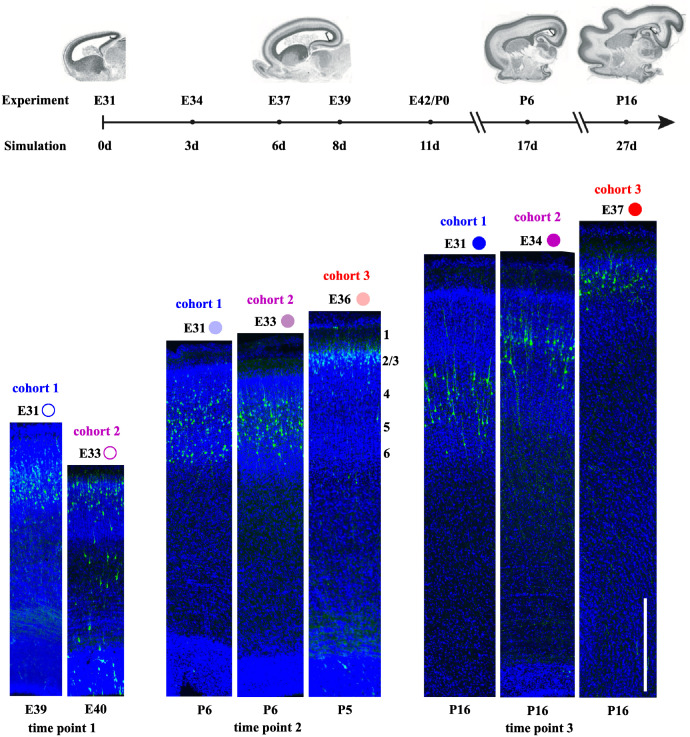
Experimental data used for calibration of the computational model. Top: time alignment between experiments and simulations. Ferrets were *in utero* electroporated between E31 and E37, and brain sections were prepared and imaged between E39 and P16; these dates correspond to simulation times between 0 and 27 days (0 d to 27 d). Images of ferret brains are reproduced from [34]. Bottom: regions of interest (ROI) taken at consistent locations in *N* = 8 typically developing ferret brains, grouped based on their imaging timepoint (E39–40, P5–6, and P16). For each timepoint, we show neuron cohorts born at different embryonic times (E31, E33–34, and E36–37) labeled with EGFP (bright green). As expected, younger neurons occupy the outermost of the cortex’s six laminae. For consistency, the data marker used in the results is shown for each of the eight samples; color indicates the cohort (cohort 1 is blue, cohort 2 is purple, and cohort 3 is red) while color saturation indicates the imaging timepoint (with lighter and darker colors representing earlier and later imaging, respectively). Scale bar, 500 μm. For detailed methods on the experimental approach, please refer to Section 4.2.

During early neurodevelopment, radial glial cells in the ventricular zone undergo multiple rounds of asymmetric cell divisions. As a result, intermediate progenitor cells are produced and then migrate into the subventricular zone, where they further proliferate to produce post-mitotic neurons (Fig 1, left). The enlarged subventricular zone, featuring inner and outer regions, serves as one of the characteristic features in mammalian brains [7]. Eventually, the neurons migrate through the intermediate zone towards the pial surface along the scaffolding of radial glial fibers. Finally, neurons accumulate at the cortical plate to form a six-layered cortex. They follow an inside-out fashion [8–10], meaning that the younger neurons bypass their older counterparts to reside near the pial surface (Fig 1, top). The early phase of neuronal migration is accompanied by a radial expansion (thickening) of the cortex, while the later accumulation of neurons results in tangential expansion of the cortical plate. The onset of cortical folding (Fig 1, right) is consistent among species, occurring after most neurons have been born and have completed their migration. In ferrets, this is approximately P6–P10 (day 6–10 after birth, where P0 is the day of birth) (Fig 2, top). Neuronal connectivity and axon formation become increasingly active around P5, and the cortical organization becomes stable one month after birth [11, 12]. The fan-like distribution of radial glial fibers induced by the buckling is consistently observed in gyrencephalic brains, which is essential for regulating neuronal migration and cortical folding [13, 14].

Multiple hypotheses of cortical folding have prevailed throughout history, and debates as to whether biology or mechanics drives cortical folding are still ongoing [12, 15]. The earliest hypothesis suggested that folding could be a passive consequence of mechanical forces acting on the expanding brain, including cerebrospinal fluid pressure and the constraints from the cranium [3]. However, experimental work by [16] showed that the forces primarily responsible for folding reside within the cortex. Nowadays, two major hypotheses are widely accepted by the community. First, the differential growth hypothesis proposed by [17] suggests that the growth rate mismatch between cortex and subcortex leads to a mechanical instability [18]. Secondly, the axonal tension hypothesis [19] suggests that the patterned axonal tension between specific cortical regions drives cortical folding. Experimental evidence has challenged these theories; in the latter case, experiments and modeling have led to a revised theory that stress-dependent axon growth and remodeling mediates, rather than drives, cortical folding [20]. However, it is likely that these phenomena work together to instigate and govern the development of cortical folds.

A number of continuum models of cortical folding (e.g. [21, 22]) have been developed to facilitate the *in silico* simulation of gyrification, leading to a deeper understanding of the forces and fundamental features involved [23]. Recently, multifield models, which couple cell density and tissue deformation, have enabled researchers to capture different aspects of brain development, including cell proliferation, migration, and diffusion, and their effects on tissue growth. The earliest papers in this field explored particularly the effects of velocity orientation [24] and cortical-subcortical stiffness and growth ratios [25]. Later, this general approach was extended to include the dependence of tissue stiffness on cell density [26] and to investigate the effect of signaling molecules on cell migration [27]. In this paper, we build on this multifield continuum framework to develop a computational model of the migration of multiple distinct cohorts of neurons in the developing brain, and to calibrate it using images of ferret brains in early development (Fig 2, bottom).

Ferrets are an ideal animal model for the study of cortical folding because of their resemblance to human brain structure and their conveniently short gestational period. Most importantly, unlike humans, where cortical folding takes place *in utero* during gestation, ferret brains fold postnatally [28, 29]. The sequence of developmental events are quite similar, although the timing varies considerably between species, primarily attributed to interspecies differences in the lengths of gestation [12, 30].

Experimental investigations of the genetic, biochemical, anatomical, and mechanical characteristics of developing brains have helped to advance our understanding of neurodevelopment and cortical folding. Although various techniques, ranging from magnetic resonance imaging to microdissection and nanoindentation [20, 31–33] have been utilized, our knowledge about the molecular mechanisms underlying formation, function, pathophysiology, and evolution is still limited. Genetic manipulation techniques, on the other hand, offer a novel means of measuring, monitoring, and modifying the process of development [35–37]. Our recent work [38] has established a method to express genes of interest in ferret neurons via *in utero* electroporation (IUE). This has several advantages over other conventional methods [39]. First, it takes only a few hours to perform on ferret embryos; ferret kits expressing transgenes can be obtained after a couple of days. Secondly, the expression of transgenes in the embryo is still detectable several months after the birth. Thirdly, the location of the transfected area can be controlled by conducting IUE at different embryonic dates. For example, transgene expression in deep cortical and superficial cortical neurons could be achieved by IUE at E31 and E37 (embryonic days 31 and 37), respectively (Fig 2, bottom). Because of these advantages and the rich experimental evidence produced, IUE is a powerful aid for the validation and calibration of theoretical and computational modeling.

This paper’s contributions are threefold. First, we refine previous multifield models of brain development [24–26] by incorporating multiple cohort neurons with neuronal migration of each cohort modeled as an advection-diffusion process. Secondly, we calibrate and validate our novel biomechanical model of neuronal migration using our innovative experimental approach for labeling and tracing neurons in the developing ferret *in vivo*. The model calibration is done via a genetic algorithm. Finally, we numerically implement the model by writing a user element subroutine (UEL) for the finite element program Abaqus/Standard (2020) [40] and provide them online for the community.

## 2 Results and discussion

First we observe and quantify the behavior of neuronal subpopulations in brain development and their effect on cortical folding; then we use this data for the calibration of a numerical model of the spatiotemporal evolution of neuron cell densities and cortical folding. Our intent is the following: 1) to understand cortical folding from a cellular perspective; 2) to demonstrate a robust numerical model of cortical folding; 3) to investigate the effect of material parameters on neuronal migration and cortical folding; and 4) qualitatively compare our model results to observed trends.

### 2.1 Experimental data captures both tissue growth and cell migration

To confirm that our experimental setup captures the known pattern of cortical development, we performed IUE experiments on ferret embryos (for detailed methods, please refer to section 4.2). We followed our well-established procedure for expressing GFP in the neurons of the ferret cerebral cortex [38, 41], and for subsequent sacrifice and imaging [42, 43]. Using IUE, we defined and tracked three distinct cohorts of neurons born at different embryonic stages. The first cohort, labeled at E31, went on to form the inner layers of the cortex (5/6), the second cohort, labeled at E33–34, formed the middle layers (3–5), and the last cohort, labeled at E36–37, formed the outer layers (2/3) [38] (Fig 2, bottom). Here we consider *N* = 8 brain sections, organized into three imaging timepoints (timepoint 1 (E39–40), timepoint 2 (P5–6), and timepoint 3 (P16)) and three cohorts of neurons, representing different IUE timepoints (cohort 1 (IUE at E31), cohort 2 (IUE at E33–34), and cohort 3 (IUE at E36–37)).

From visual inspection of the resulting image, we see that the distance from the subventricular zone to the cortical plate increases from timepoint 1 to 3 (Fig 2, bottom). Averaging across all images in each timepoint, the lengths increased from $l_{t1}^{\text{exp}}=1390\mu \text{m}$ to $l_{t2}^{\text{exp}}=1997\mu \text{m}$ to $l_{t3}^{\text{exp}}=2390\mu \text{m}$.

Furthermore, the GFP-positive neurons, representing the cohort of neurons that was labeled by IUE at a given time, are seen to occupy successively further regions of the cortical plate. This is in agreement with the known pattern of cortical formation, where younger neurons bypass older neurons to form outer layers [8–10].

Here we note that the small peaks seen in the experimental data for cohorts 1 and 2 at the first timepoint, which are not predicted by our model, represent GFP-positive fibers, not neurons, and should be ignored.

### 2.2 Radial cell density profiles show that younger neurons form successive outer layers of the cortex

**Fig 3:**
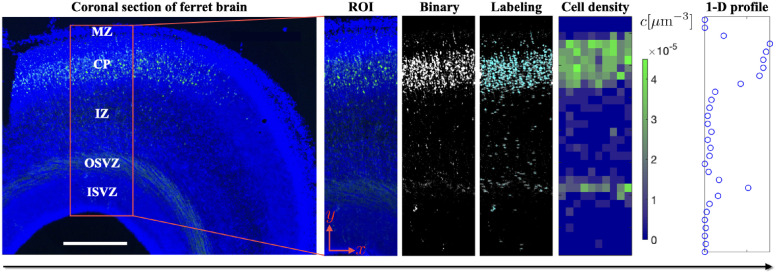
Workflow of cell density calculation. The ferret brain sections could be divided into five anatomical zones: marginal zone (MZ), cortical plate (CP), intermediate zone (IZ), outer subventricular zone (OSVZ), and inner subventricular zone (ISVZ). First, we select an ROI aligned radially, such that cell migration along radial glial fibers consistently occurs along the length of the domain. Secondly, the high-resolution image is imported into ImageJ [44] and neurons are segmented based on the color threshold. Each neuron and its coordinates are then labeled and recorded automatically. Thirdly, we post-process the data in Matlab to generate a neuron density plot by counting neurons in each subcell. Finally, the cell density profile along the y-axis is generated by averaging the data in the x-axis direction. Here we note that the small peak seen in the OSVZ represents GFP-positive fibers, not neurons. Scale bar, 500 μm. For detailed methods on the image analysis, please refer to Section 4.3.

To gather experimental data for our model calibration we analyzed images resulting from our IUE experiments. From each image, we selected a region of interest (ROI) consisting of a rectangular region spanning from the inner subventricular zone to the marginal zone, whose length coincides with the radial direction of the brain, such that neuronal migration is dominant in only one direction (Fig 3). We then used ImageJ [44] to count the GFP-labeled neurons, calculate their density at each location, average this density across the width of the region, and normalize it along the length, to determine a one-dimensional radial cell density profile (Fig 3, right). Note that while the profile only varies along the radial position, we determined the cell density per unit volume by using information about the width and the assumed depth of the image. Collectively, these data capture both the overall expansion of the brain and the careful inside-out arrangement of neurons, from oldest to youngest, in the cortex. These data were used for our model calibration.

### 2.3 Our model captures the behavior of three distinct neuronal populations

To capture the behavior seen in our experimental data, we extend previous multifield models [24–26] to consider three cell types, here representing cohorts of neurons electroporated at a specific time, and track their cell density *c*_*i*_(**x**, *t*) as a function of space and time (please refer to Section 4.4 for detailed methods). The cell density changes as a result of neurogenesis, cell migration, and volume change. Neurogenesis varies spatiotemporally, while cell migration, volume growth, and mechanical properties vary spatially throughout the domain, governed by a set of parameters (Table 1).

**Table 1:** Summary of material parameters.

**predefined parameters**			
parameter [units]	value	source	
threshold cell density *c*_0_ [μm^−3^]	1.0 × 10^−6^		
sensitivity of activation *α*_*c*_ [−]	0.1		
initial radius *R*_0_ [μm]	239.0	[34]	
length of ventricular zone *δ*^*x*^ [μm]	0.2*R*_0_	[25]	
smoothing parameter *α*_*G*_ [−]	0.05		
electroporation time $\delta_{i}^{t}\;\left[\text{d}\right]$	[0, 3, 6]	protocol	
smoothing parameter *ϵ* [d]	2.0		
smoothing parameter *α*_*v*_ [−]	0.05		
tangential-radial growth ratio *β*_*k*_ [−]	1	[25]	
location of growth mode transition *δ*_*k*_ [μm]	0.93*R*_0_		
smoothing parameter *α*_*k*_ [−]	1.0		
subcortical shear modulus *μ*_*s*_ [kPa]	1.0	[45]	
Poisson’s ratio *ν* [−]	0.45	[45]	
stiffness ratio *β*_*μ*_ [−]	3	[46]	
location of stiffness transition *δ*_*μ*_ [μm]	0.93*R*_0_		
smoothing parameter *α*_*μ*_ [−]	1.0		
**calibrated parameters**			
parameter [units]	value	source	range
baseline division rate constant *G*^*c*^ [μm^−3^ d^−1^]	1.41 × 10^−5^	[25]	1.0 × 10^−6^–1.5 × 10^−5^
baseline velocity constant *v*_*i*_ [μm d^−1^]	1472.2	[47]	230.0 − 4752.0
final destination $\delta_{i}^{v}\;\left[\mu \text{m}\right]$	[191.6, 210.7, 222.7]		
diffusion coefficient *D* [μm^2^ d^−1^]	31612.6	[48]	25000.0–35000.0
subcortical growth parameter *k*_*s*_ [μm^3^]	202950		

**Table 2:** Comparison of parameters between the original three-cohort model calibrated to the full set of experimental data, the model validation where the third timepoint was omitted, and the single-cohort model. Percent differences relative to the original three-cohort model for the latter two models are shown. *The single-cohort model only has a single destination parameter, *δ*^*v*^; here we compare it individually to the three separate destination parameters from the three-cohort model.

parameter	three-cohort model	model validation		single-cohort model	
	value	value	% diff.	value	% diff.
*G*^*c*^ [μm^−3^ d^−1^]	1.41 × 10^−5^	1.4 × 10^−5^	0.7	3.3 × 10^−5^	134.0
*v*_*i*_ [μm d^−1^]	1472.2	1553.4	5.5	1473.8	0.1
$\delta_{1}^{v}\;\left[\mu \text{m}\right]$	191.6	199.9	4.3	209.4*	9.2
$\delta_{2}^{v}\;\left[\mu \text{m}\right]$	210.7	207.9	1.3	209.4*	0.6
$\delta_{3}^{v}\;\left[\mu \text{m}\right]$	222.7	222.9	0.8	209.4*	5.9
*D* [μm^2^ d^−1^]	31612.6	25059.2	20.7	34600.7	9.4
*k*_*s*_ [μm^3^]	202950	215408	6.1	244743	20.5

To determine the best parameters to represent ferret neurodevelopment, we calibrate our model against the cell density profiles obtained from experimental images (Fig 2, bottom). We considered a three-dimensional slender bar in our simulation to represent the experiment best; as an approximation, we let results of this simulation only vary along the length of the bar (i.e. a 1-D solution). This was chosen for its simplicity and its compatibility with the definition of the model parameters (e.g., per unit volume).

**Fig 4:**
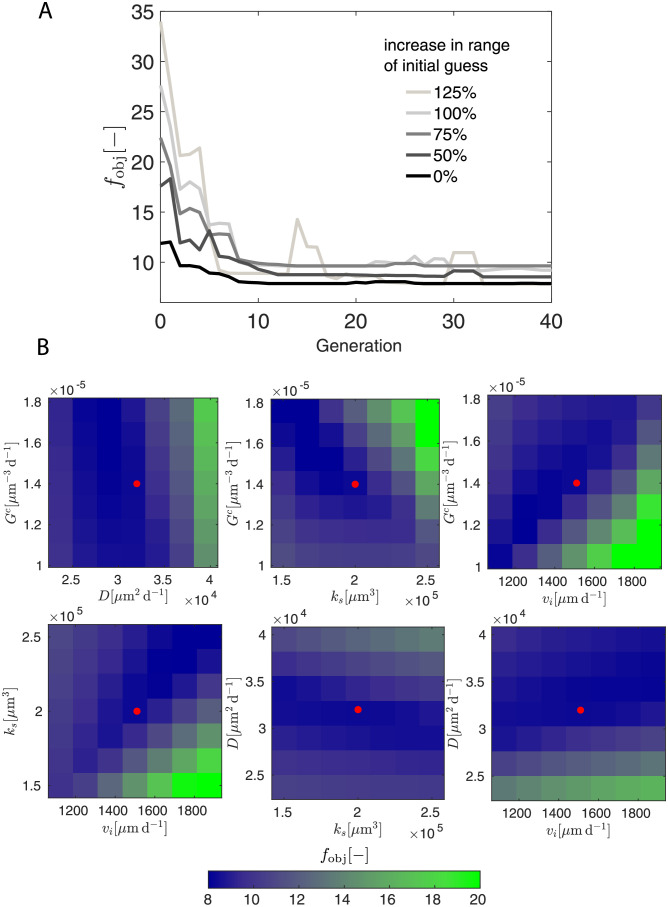
Convergence and sensitivity analysis of model calibration. A) the objective function *f*_obj_ as a function of generation. B) sensitivity study of objective function *f*_obj_ with respect to baseline division rate constant *G*^*c*^, diffusion coefficient *D*, baseline velocity constant *v*_*i*_, and subcortical growth parameter *k*_*s*_. The red dots in each 2-D parameter space denote the calibrated parameter set.

For calibration, we use a genetic algorithm—an adaptive heuristic search algorithm capable of handling optimization problems with highly nonlinear, discontinuous, and multi-purpose objective functions. The genetic algorithm borrows the idea of natural selection and gene evolution from biology, including inheritance, selection, crossover, and mutation [49]. We define an objective function, *f*_obj_, which effectively sums the errors in displacement and cell density between the simulations and experimental data (please refer to Section 4.6 for more details). We obtain the optimal set of parameters when the objective function *f*_obj_ is minimized and no longer altering with the generation (Fig 4A).

**Fig 5:**
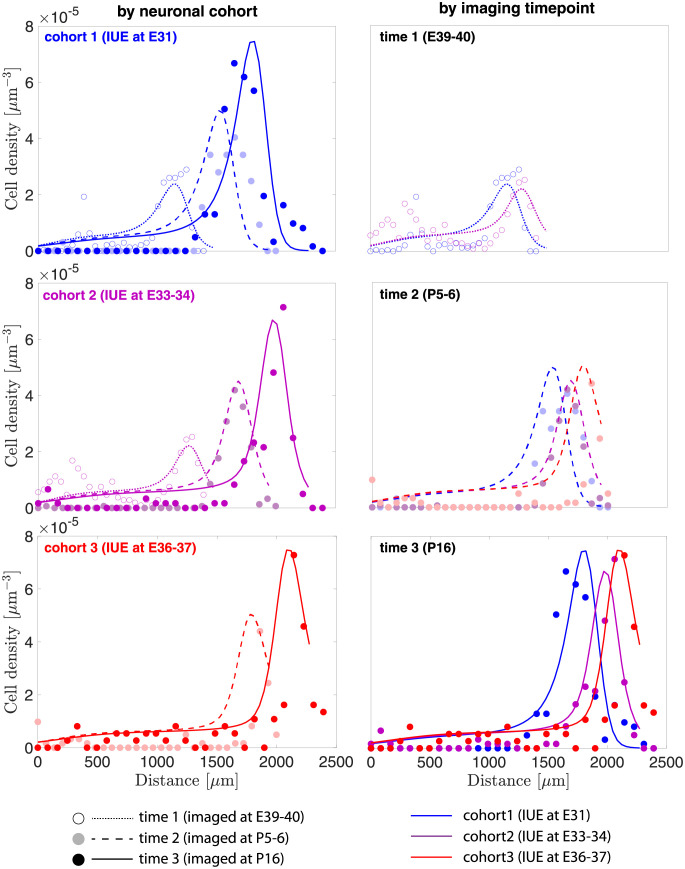
Neuronal cell density as a function of distance from the subventricular zone, compared between experiments (circles) and simulations (lines). Results are organized both by neuronal cohort (left column) and imaging timepoint (right column). Note that colors represent neuronal cohorts based on IUE dates, while different markers and line styles differentiate imaging timepoints.

Here we focus on calibrating seven material parameters: the baseline division rate *G*^*c*^, which affects the proliferation of neurons in the subventricular zone; baseline velocity *v*_*i*_, which describes the velocity of migrating neurons throughout their journey; final destination $\delta_{i}^{v}$, which defines each cohort’s terminal location in the cortical plate; diffusion coefficient *D*, which controls the neurons’ tendency to spread out; and subcortical growth constant *k*_*s*_, which describes the volumetric growth in the subcortex. When based on calibrated material parameters (Table 1), our results show a good agreement between experiments and model (Fig 5), particularly by capturing both the overall change in the tissue geometry due to growth, and also the specific behavior of each of the three neuronal cohorts.

### 2.4 Our model predicts P16 results based on data from E39–40 and P5–6 timepoints

**Fig 6:**
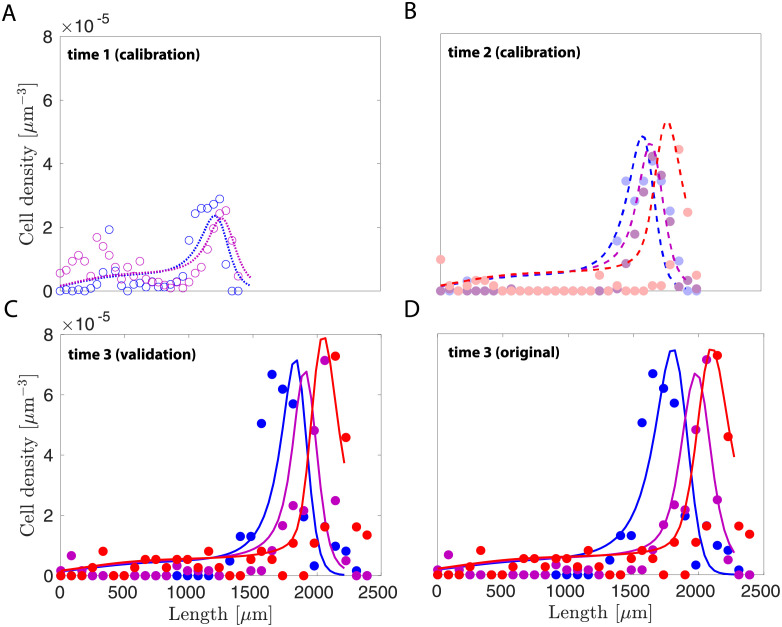
Model validation via leave-one-out approach. Our model was first calibrated to time points of A) E39-40 and B) P5-6, then used to predict the third time point of C) P16. D) The original model calibration at time 3 for the sake of comparison.

For the purpose of validating our model, we adopted a leave-one-out approach—calibrating the model to incomplete data first and then using the rest of the data for validation. Here, we focus on validating time predictions for our model. First, the model was calibrated to the first two imaging time points of E39–40 and P5–6, and then we compared the model’s prediction with the third time point of P16 (Fig 6A–6C). The original calibration at time 3 was included for the sake of comparison (Fig 6D). We also compared the calibrated parameter set with the original set in Table 2. It shows that the new set is similar to the original set and our model can predict the data reasonably well given incomplete data in time.

### 2.5 A three-cohort model largely mimics a single-cohort model, with additional microscale resolution

**Fig 7:**
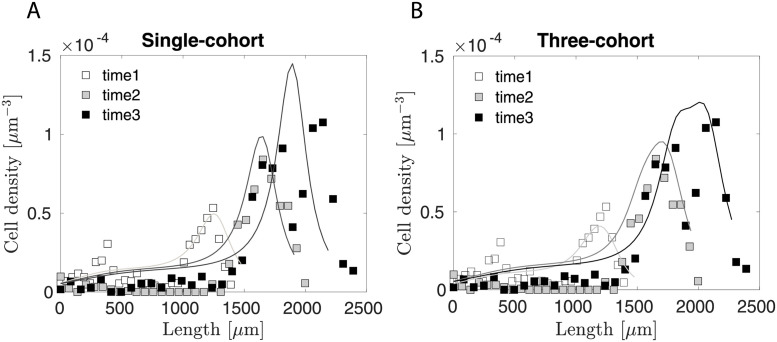
Comparison between A) the single-cohort model and B) the three-cohort model in capturing the experimental data. In both cases, data points represent summed cell densities across all three neuronal cohorts, $c=\sum_{i=1}^{3}c_{i}$. For comparison, the total cell density of the three-cohort model is calculated similarly, representing the sum of the different colored lines in Fig 5, right.

To highlight the superiority of the multi-cohort over the single-cohort model in capturing the experimental data, we simplified our model to account for a single neuronal cohort, which was calibrated to data that sums up all three neuronal cohorts (Fig 7A). The calibration shows that the single cohort model could capture the data reasonably well except for the spatial distribution of cell density in time point 3, which the three-cohort model is capable of capturing (Fig 7B). We also compared the calibrated parameters with the original set in Table 2. Note that the baseline division rate constant increases over twofold, which is expected as the same number of cells have to be produced over the same period. The diffusivity is also increased to make the cell more spread out in space, a feature that naturally results from our three-cohort model.

### 2.6 Our model calibration is robust to a large increase in parameter ranges

The convergence of a genetic algorithm is case-sensitive and strongly depends on how far the initial guess is away from the optimized parameter set. For this reason, we restricted some of the calibrated parameters a range of reasonable values based on the literature. In particular, the baseline rate constant *G*^*c*^ has been found to be between 1 × 10^−6^ − 15 × 10^−6^μm^−3^ d^−1^ [25], the diffusion coefficient *D* between 25000–35000 μm^2^ d^−1^ [48], and the baseline velocity constants *v*_*i*_ between 230–4752 μm d^−1^ [47].

To determine the sensitivity of our calibration to the initial guess, we experimented with larger bounds for this range of reasonable values. We reran the calibration several times, increasing the initial range for each parameter, within which the initial guesses are generated randomly, by 50%, 75%, 100%, and 125% (Fig 4A). All runs converged to very similar values within the original range. As we expected, a larger parameter bound makes it more difficult for the genetic algorithm to converge (Fig 4A). This suggests that the ranges found in the literature reflect the physiological bounds of these quantities, as even random initial guesses within those bounds reflect our experimental data better than cases where the limits were expanded, even after multiple generations. We also present the details of the parameters’ evolution as a function of the genome in S1 Appendix. Overall, our results show that the genetic algorithm is robust for solving our complex optimization problem.

### 2.7 Our model is most sensitive to neuronal proliferation and velocity

In order to determine which model parameter most strongly influence our model predictions, we performed a sensitivity study. In particular, we investigated the sensitivity of our objective function to the model parameters of baseline division rate constant *G*^*c*^, diffusion coefficient *D*, baseline velocity constant *v*_*i*_, and subcortical growth parameter *k*_*s*_ (Fig 4B). Our results suggest that the calibrated sets are mainly located at the local minimum in all subplots. Moreover, the objective function *f*_obj_ is strongly sensitive to the baseline division rate constant *G*^*c*^ and baseline velocity constant *v*_*i*_, moderately sensitive to subcortical growth parameter *k*_*s*_, and mildly sensitive to the diffusion coefficient *D*.

### 2.8 Our simulations qualitatively recapitulate the typical development of the ferret brain

In order to understand the cortical folding behavior predicted by our model, we simulated the typically developing ferret brain with the calibrated parameters obtained previously. Note that we do not intend to have a quantitative full-field comparison between our cortical folding simulations and real ferret brains, but rather present a qualitative study based on a reasonable set of parameters.

**Fig 8:**
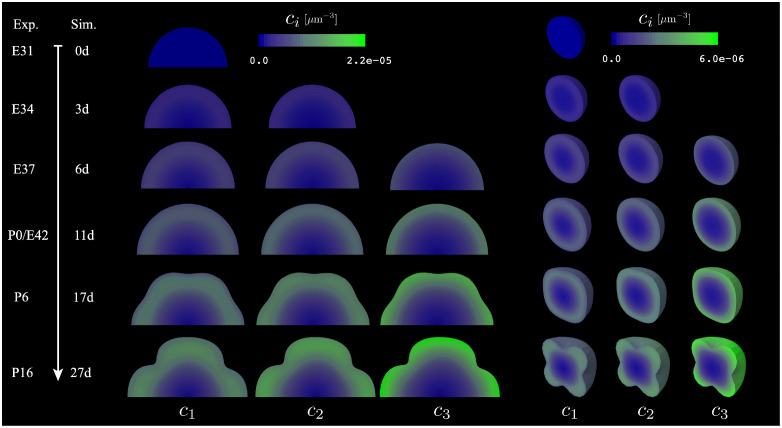
2-D and 3-D simulations of cortical folding from 0d to 27d (corresponding to E31 to P16 in ferret development), with contour plots showing cell density *c*_*i*_ for three cohorts of neurons. Note that the time between shown timepoints is not consistent, as timepoints were selected on the basis of biological relevance.

Our simulations are qualitatively in line with our experiments (Fig 8). As expected, cohorts of neurons generated at E31, E33-34, and E36-37 gradually reside at the cortex’s lower, medium, and upper layers, respectively. Furthermore, the onset of mechanical buckling is around P6, when cortical folding begins in healthy ferret brains [32]. While these results are similar to those seen in previous work [24–26], our model additionally contains information on the behavior of three distinct neuronal populations. In the future, we expect this difference to be even more important as we expand into the study of abnormal development, where spatiotemporal changes in neuron behavior (for example, due to a local injury or a drug exposure at a certain point in gestation) could be modeled and their effect on brain development predicted.

### 2.9 Stiffness ratio and tangential-radial growth ratios affect the onset of buckling and resulting wavelength

**Fig 9:**
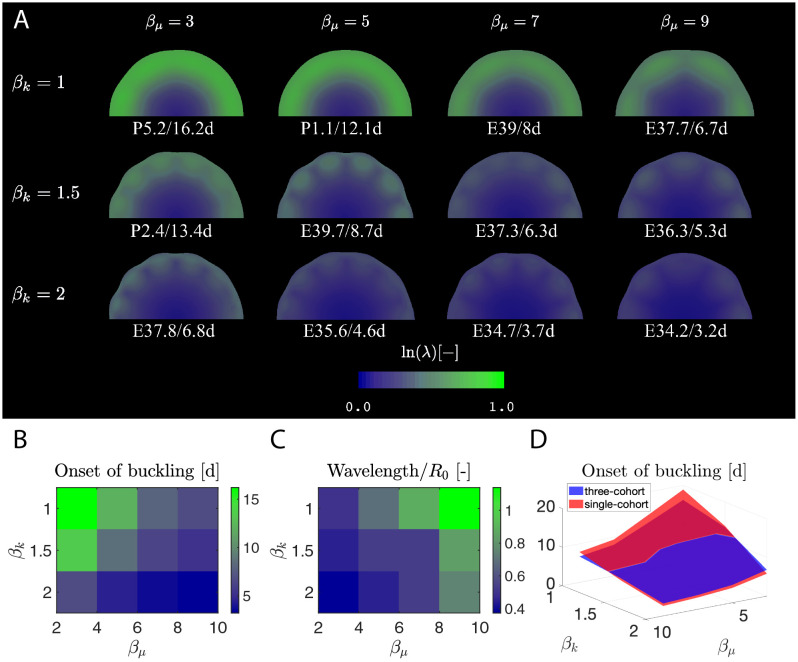
Factors that influence buckling point and wavelength in 2-D simulations. The cortical-subcortical stiffness ratio *β*_*μ*_ and tangential-radial growth ratio *β*_*k*_ influence the contour plots of A) true strain ln(λ), B) the onset of buckling, and C) the normalized wavelength. Note that each simulation shown in A) is taken at the buckling point. D) The three- and single-cohort models predict similar buckling points, with only small differences.

In order to understand the effects of the gray-white stiffness ratio *β*_*μ*_ and the tangential-radial growth ratio *β*_*k*_, we varied these parameters around their initial assumed values. Specifically, we consider different combinations of *β*_*μ*_ = [3, 5, 7, 9] and *β*_*k*_ = [1, 1.5, 2] (Fig 9). Note that each contour plot of true strain ln (λ) in Fig 9A is captured at the buckling point. We additionally show the buckling point quantitatively (Fig 9B), showing that buckling occurs earlier as both stiffness ratio *β*_*μ*_ and growth ratio *β*_*k*_ increase—that is, when the cortex is relatively more stiff compared to the subcortex, and when tangential growth dominates more over radial growth in the cortex. We also measured the wavelength of each simulation and normalized it by the initial radius *R*_0_. It shows that the wavelength correlates positively with stiffness ratio *β*_*μ*_ and negatively with the tangential-radial growth ratio *β*_*k*_ (Fig 9C). Moreover, crease formation is more energetically favorable at a smaller stiffness ratio *β*_*μ*_, as is reported by the literature [18, 46, 50–52]. Finally, we made a comparison between the three-cohort model with the single-cohort one in terms of buckling point, showing that they share a similar trend when calibrated to the same set of experimental data Fig 9D).

### 2.10 Radial glial fiber distribution and orientation occurs naturally from cortical folding

**Fig 10:**
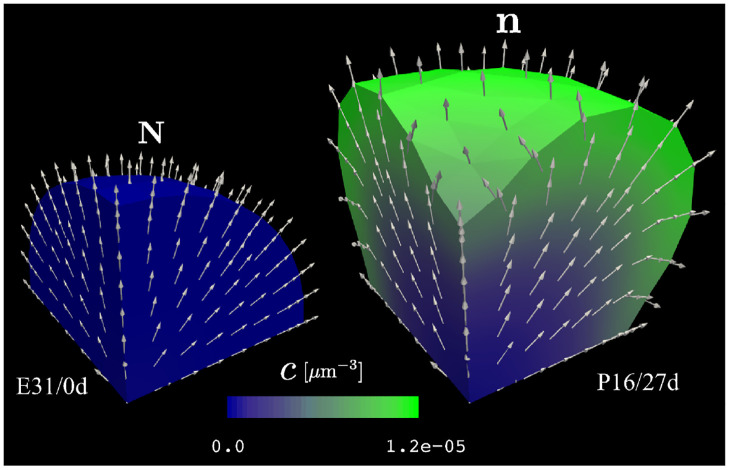
Simulated 3-D radial glial fiber orientations at 0d and 27d with contour plots showing total cell density *c*.

By visualizing the direction of the deformed radial vector **n**, we can see how radial glial fibers, which are initially aligned perfectly radially along unit vector **N**, deform through the process of cortical folding. In our simulations, we see a fan-like distribution of these fibers, diverging drastically towards the pial surface in gyri (Fig 10). This is of interest because this distribution is consistently observed in gyrencephalic species and is vital in distributing neurons within the cortex [13]. It has even been proposed that the orientation and distribution of radial glial fibers regulate cortical folding [13, 14]. Our simulations show that the fan-like distribution of radial glial fibers arises naturally from the mechanical instabilities.

## 3 Concluding remarks

In this work, we have developed a biomechanical model for numerical simulations of brain development. The current study extends pioneering multifield models of brain development [24, 25]. Our proposed model extends this earlier work to account for the spatial-temporal development of multiple cohorts of neurons, each modeled as an advection-diffusion process. The model was implemented numerically by writing customized finite elements in the commercial finite-element program Abaqus/Standard (2020) [40]. The user-element subroutines, as well as representative Abaqus input files, are available online (https://github.com/mholla/neuronal_migration). We also conducted novel experiments on ferret brains via IUE, which was used for model calibration. We have studied typical cortical folding in ferret brains by integrating experiments and numerical simulation capabilities. Our simulations qualitatively agree with the experiments on ferret brains.

Our results suggest significant opportunities for further extensions and improvements in future work. First, the current work accounts for only three cohorts of neurons for simplicity, while in reality, neurons are continuously generated within the ventricular zone. There are also other components of brain tissue, such as glial cells, that are also believed to play an important role in brain development [53]. Secondly, we assumed constant mechanical properties throughout the brain. However, brain tissue exhibits rich and complex mechanical interactions, such as the tendency for axons to grow under tension [54], and cells to migrate following mechanical cues such as the gradient of stiffness and stress state [55]. These features are not yet captured in our model. Finally, experiments of genetic manipulation via IUE have shown the important role of a number of genes in the normal development of cortical folds [56–58], and that disruption of these genes can drastically alter the resulting brain morphology. In particular, we have shown that altering deep and superficial neurons leads to different effects [56]. Our model is uniquely capable of capturing the effects of neuronal subpopulations on cortical folding. Thus, our model could enable the study of spatiotemporal events in neurodevelopment, where the behavior of some neuronal population is changed by an injury, exposure, or treatment at a specific location or time during development. For instance, our previous experiments have shown that the loss of gene Cdk5, which is expressed in post-mitotic neurons, affects upper-layer neurons (like our cohort 3) differently than in inner-layer neurons (like our cohort 1) [56]; folding proceeds normally in the latter case, while the former results in impaired cortical folds in ferrets. This behavior can’t be captured in a single cohort-model, where all neurons are grouped in a single population with the same behaviors. In the future, our model can be adapted and calibrated to this experimental, and used for the design of new experiments, *in silico* testing of mechanistic hypotheses, and perhaps even predictions of effective treatments. Adapting our computational framework to account for these features will be the focus of future work.

## 4 Materials and methods

### 4.1 Ethics statement

We purchased normally pigmented sable ferrets (*Mustela putorius furo*) from Marshall Farms (North Rose, NY). Ferrets were maintained as described in our previous works [38, 59, 60]. All procedures were performed following a protocol approved by the Animal Care Committee of Kanazawa University.

### 4.2 Experimental methods

We followed our well-established procedure for IUE to express transgenes in the neurons of the ferret cerebral cortex [38, 41]. First, pCAG-GFP was prepared according to the protocols used in our previous work [61]. Plasmids were purified using the Endofree plasmid Maxi kit (Qiagen, Valencia, CA). Before IUE, plasmid DNA was diluted to 0.5 − 3.0 mg mL^−1^ in 1× phosphate-buffered saline (PBS), and Fast Green solution was added to a final concentration of 0.5% to monitor the injection. While body temperature was maintained with a heating pad, the uterine horns of pregnant ferrets were exposed and kept wet by adding drops of PBS intermittently. The location of embryos was visualized with transmitted light delivered through an optical fiber cable. The pigmented iris was visible, and this enabled us to assume the location of the lateral ventricle. Approximately 2 μL to 5 μL of DNA solution was injected into the lateral ventricle at the indicated ages using a pulled glass micropipette. Each embryo within the uterus was placed between tweezer-type electrodes with a diameter of 5 mm (CUY650-P5; NEPA Gene, Japan). Square electric pulses (50–150 V, 50 ms) were passed five times at one-second intervals using an electroporator (ECM830, BTX). Care was taken to quickly place embryos back into the abdominal cavity to avoid excessive temperature loss. We then sutured the wall and skin of the abdominal cavity and allowed embryos to develop normally.

After IUE, ferrets were sacrificed and imaged at various embryonic ages and postnatal dates, following the procedure used in our previous works [42, 43]. Animals were deeply anesthetized and transcardially perfused with 4% paraformaldehyde (PFA) before brain removal. The brains were then cryoprotected by overnight immersion in 30% sucrose and embedded in OCT compound. Sections of 50 μm thickness were incubated with 1 μg mL^−1^ Hoechst 33342, washed, and mounted before epifluorescence imaging.

### 4.3 Image analysis

Here we consider *N* = 8 brain sections, covering different IUE dates corresponding to distinct “cohorts” of neurons, and imaging timepoints (Fig 2, bottom). We organized them into three imaging timepoints: timepoint 1 (E39-40), timepoint 2 (P5-6), and timepoint 3 (P16). We also organized them into three IUE groups, each representing a different cohort of neurons: cohort 1 (IUE at E31), cohort 2 (IUE at E33-34), and cohort 3 (IUE at E36-37).

We considered a region of interest (ROI) in each brain, consisting of a rectangular region spanning from the marginal zone to the inner subventricular zone, in which the neuronal migration is dominant in one direction (y-axis) (Fig 3).

We imported high-resolution images of each ROI into ImageJ [44] for cell counting. For consistency, the length of each ROI was averaged within each imaging group ($l_{t1}^{\text{exp}}=1390\mu \text{m}$, $l_{t2}^{\text{exp}}=1997\mu \text{m}$, and $l_{t3}^{\text{exp}}=2390\mu \text{m}$) and location data were normalized by this length. The neurons were segmented by converting the images into a binary format based on the color threshold (Fig 3). Each neuron was then labeled, and its location was recorded automatically via a built-in particle analysis function in ImageJ [44]. Next, the data containing neuron coordinates were imported into Matlab for cell density calculation. We divided each ROI into numerous subcells with a dimension of 45.1 μm × 49.3 μm × 50 μm and then counted the number of neurons within each subcell accordingly (Fig 3). Since we restrict our attention to cell density along the y-axis, the cell density across the x-axis is averaged for the final plot. We repeated the process on all *N* = 8 ferret brains to obtain the 1-D cell density profiles, and this set of data was used for our model calibration (Fig 5, circles).

### 4.4 Mathematical model

#### 4.4.1 Kinematics

Consider a body $B_{\text{R}}$ identified with the region of space it occupies in a fixed reference configuration, and denote by **x**_R_ an arbitrary material point of $B_{\text{R}}$. The referential body $B_{\text{R}}$ then undergoes a motion $x=\chi (x_{\text{R}},t)$ to the current deformed body $B_{t}$ with deformation gradient given by

$$\begin{array}{c} F=\nabla \chi ,\;\text{such}\;\text{that}\;J=\text{det}\;F>0\;, \end{array}$$ (1)

where ∇ denotes the gradient with respect to the material point **x**_R_ in the reference configuration. To take growth-related changes in volume within the region into account, we follow [62] in adopting the multiplicative decomposition of the total deformation gradient,

$$\begin{array}{c} F=F^{e}F^{g}, \end{array}$$ (2)

where **F**^g^ is the irreversible growth part of the deformation measuring from reference configuration $B_{\text{R}}$ to the intermediate stress-free configuration $B_{g}$ denoted by $\bar{x}$, while **F**^e^ is the reversible elastic part of the deformation measuring from the intermediate to the current configuration $B_{t}$ (Fig 1). Similarly, the volumetric change *J* can be decomposed into elastic and growth part, i.e.,

$$\begin{array}{c} J=\text{det}\;F=J^{e}J^{g}. \end{array}$$ (3)

Unlike most phenomenological models of growth, here we will define the growth tensor **F**^g^ as a function of local neuron density; its form will be specified in Section 4.4.4. The elastic tensor can then be found as **F**^e^ = **FF**^g−1^, and the elastic left and right Cauchy-Green tensors are given by

$$\begin{array}{c} B^{e}=F^{e}{F^{e}}^{\;⊤}\;\text{and}\;C^{e}={F^{e}}^{\;⊤}F^{e}. \end{array}$$ (4)

#### 4.4.2 Balance of cell density

Previous models of coupled cell density and volume growth have considered a single parameter that describes the number of cells per volume throughout the domain [24–26]. Here, to align with our experiments, we extend the model to consider three cell types, representing cohorts of neurons electroporated at a specific time point. We define the spatial cell density *c*_*i*_(**x**, *t*) as the number of cells in the *i*th cohort per unit current volume. The referential form of balance of cell density for each cohort of neurons in an undeformed body $B_{\text{R}}$ is given by ${\dot{c}}_{0i}=F_{i}^{c}+\text{Div}\;Q_{i}$, where *c*_0*i*_ = *Jc*_*i*_ is the referential cell density, $F_{i}^{c}$ and **Q**_*i*_ are cell source and cell flux in reference state, respectively. We now rewrite the balance of cell density in the spatial form,

$$\begin{array}{c} {\dot{c}}_{i}+c_{i}\frac{\dot{J}}{J}=f_{i}^{c}+\text{div}\;q_{i}\;, \end{array}$$ (5)

where all referential quantities are written in terms of their spatial counterparts, i.e., $F_{i}^{c}=Jf_{i}^{c}$, **Q**_*i*_ = *J***F**^−1^**q**_*i*_. It is worth noting that the second term on the left-hand side in Eq (5) is the change rate of cell densities associated with the volumetric changes. We will specify spatial cell source $f_{i}^{c}$ and spatial cell flux **q**_*i*_ later in Section 4.4.4.

#### 4.4.3 Equilibrium

Neglecting inertial effects and body forces, the balance of forces and moments in the deformed body $B_{t}$ are given by

$$\begin{array}{c} \text{div}\;T=0\;\text{and}\;T=T^{\;⊤}, \end{array}$$ (6)

respectively, where **T** is the Cauchy stress. The external surface traction on an element of the deformed surface $\partial B_{t}$ is given by

$$\begin{array}{c} \overset{˘}{t}\left(n\right)=\left[\left[T\right]\right]n, \end{array}$$ (7)

where [[**T**]] = **T**^in^ − **T**^out^ across the boundary $\partial B_{t}$, and **n** denotes the outward surface normal at the boundary.

#### 4.4.4 Constitutive equations

Here we specify the constitutive equations for neurogenesis, neuron density flux, growth deformation kinematics, and mechanical free energy.

**Neurogenesis**. Unlike previous work [25, 26], in which the cell source is only a function of space, we add a time component to represent the neurons that are affected by IUE performed at a given timepoint, i.e.,

$$\begin{array}{c} f_{i}^{c}=G^{c}G^{x}\left(x_{\text{R}}\right)G_{i}^{t}\left(t\right), \end{array}$$ (8)

where *G*^*c*^ is the baseline division rate constant, *G*^*x*^(**x**_R_) is the spatial distribution, and $G_{i}^{t}\left(t\right)$ is the temporal distribution for *i*th cohort of neurons.

**Neuron density flux**. Following [25], the neuron density flux **q**_*i*_ for each cohort of neurons in Eq 5 is an overall consequence of cell advection and diffusion,

$$\begin{array}{c} q_{i}=\underset{\text{advection}\;\text{term}}{\underset{︸}{-c_{i}\hat{H}\left(c_{i}-c_{0};\alpha_{c}\right)v_{i}\left(x\right)}}+\underset{\text{diffusion}\;\text{term}}{\underset{︸}{D\cdot \text{grad}\;c_{i}}}, \end{array}$$ (9)

where the advection term on the right-hand side characterizes how neurons migrate along a specific direction, while the diffusion term describes how neurons spread out in space.

**Fig 11:**
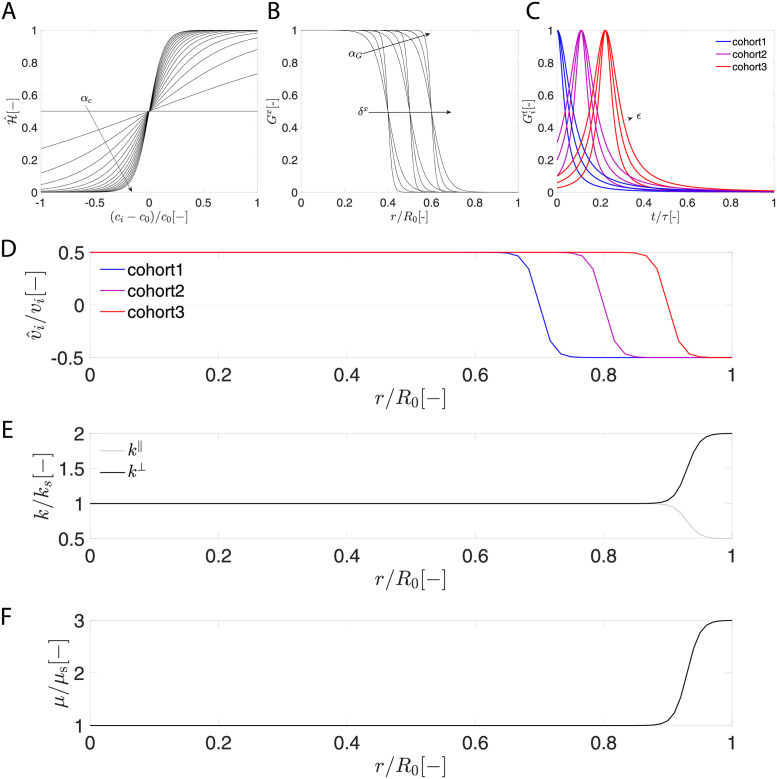
Distributions of model parameters. A) Heaviside function $\hat{H}$ as a function of normalized cell density (*c*_*i*_ − *c*_0_)/*c*_0_, B) Heaviside function *G*^*x*^ as a function of normalized distance *r*/*R*_0_, C) delta function $G_{i}^{t}$ as a function of normalized time *t*/*τ*, D) the normalized velocity profile ${\hat{v}}_{i}/v_{i}$, E) the normalized coupling parameter *k*/*k*_*s*_ profile, and F) the normalized shear modulus *μ*/*μ*_s_ profile.

First, as to the advection term, the activation of neuronal migration is controlled by a smoothed Heaviside function [26],

$$\begin{array}{c} \hat{H}\left(c_{i}-c_{0};\alpha_{c}\right)=\frac{e^{\alpha_{c}\left(c-c_{0}\right)}}{1+e^{\alpha_{c}\left(c-c_{0}\right)}}, \end{array}$$ (10)

where *c*_0_ and *α*_*c*_ are threshold value and sensitivity parameter, respectively. The neuronal migration is halfway activated when the current cell density *c*_*i*_ reaches the threshold value *c*_0_, and parameter *α*_*c*_ controls the sensitivity of activation to the cell density (Fig 11A). The magnitude and direction of the neuronal migration are characterized by a velocity field

$$\begin{array}{c} v_{i}\left(x\right)={\hat{v}}_{i}\left(x\right)n/\|n\|, \end{array}$$ (11)

where ${\hat{v}}_{i}\left(x\right)$ is the velocity magnitude, and **n** is the direction of the glial fibers in the current state, **n** = **FN** (Fig 1).

Next, as to the diffusion term, we adopt the standard Fick’s first law, in which the cell density flux depends linearly on the cell density gradient. Additionally, the diffusivity tensor **D** is assumed to have a spherical form, i.e., **D** = *D***1** where *D* denotes the diffusion coefficient.

**Growth kinematics**. According to the literature [63, 64], neurons that migrate along glial fibers (defined in our model as radial unit vector **N**) at the early stage contribute to radial expansion, while the tangential expansion in the cortical plate occurs later when neurons reach their destinations. Thus, we take the growth deformation gradient as a linear combination of radial and tangential deformation [25],

$$\begin{array}{c} F^{g}=\vartheta^{\|}\left(c\right)\left(N⊗N\right)+\vartheta^{⊥}\left(c\right)\left(1-N⊗N\right), \end{array}$$ (12)

where *ϑ*^∥^(*c*) and *ϑ*^⊥^(*c*) are independent scalar growth parameters that describe the radial growth parallel to and tangential growth normal to the glial fiber direction **N** (Fig 1). The coupling between growth parameters and cell density are given by

$$\begin{array}{c} \vartheta^{\|}\left(c\right)=1+k^{\|}c\;\text{and}\;\vartheta^{⊥}\left(c\right)=1+k^{⊥}c, \end{array}$$ (13)

where *k*^∥^ and *k*^⊥^ are radial and tangential growth parameters. Note that $c=\sum_{i=1}^{3}c_{i}$ in Eqs (12) and (13) is the *total* cell density.

**Mechanical free energy**. The ferret brain is modeled as standard neo-Hookean material, as is the case for most of the literature [46, 50–52],

$$\begin{array}{c} \psi_{\text{R}}\left(C^{e},J^{e}\right)=\frac{\mu }{2}[\text{tr}\;\left(C^{e}\right)-3-2\;\text{ln}\left(J^{e}\right)]+\frac{L}{2}\;{\text{ln}}^{2}\left(J^{e}\right), \end{array}$$ (14)

where *μ* and *L* denote Lamé constants, which may vary spatially, and only elastic deformation induces stress. The Cauchy stress is thus given by

$$\begin{array}{c} T=\frac{2}{J^{e}}F^{e}\frac{\partial \psi_{\text{R}}}{\partial C^{e}}F^{e\;⊤}=\frac{1}{J^{e}}[\mu B^{e}+(L\;\text{ln}(J^{e})-\mu )1]. \end{array}$$ (15)

#### 4.4.5 Model description and parameter distributions

For calibration of our model, we first simulate cell migration and tissue deformation along a slender bar similar to the ROI used in our experimental analysis. Then, we simulate cortical folding in a plane-strain half circle and a one-eighth sphere. Each domain has an initial radius of *R*_0_ = 239 μm, which is an averaged value measured from ferret brains sacrificed at E31 [34]. All of our simulations cover a duration of *τ* = 27 d starting from E31, chosen as it coincides with the earliest day of the experimental procedure (Fig 2, top). We begin with a stress-free state with no neurons, and we follow three cohorts of neurons generated at *t* = [0, 3, 6] d migrating from the ventricular zone to their destinations in the cortical plate.

The properties of the model vary temporally and spatially, with their distributions described below. Note that distributions only vary radially; because of this, the slender bar yields a 1-D solution, the plane-strain half circle yields a 2-D solution, and the partial sphere leads to a fully 3-D solution. For simplicity, we refer to them as 1-D, 2-D, and 3-D models, although it should be noted that the geometry in each is three dimensional and the meaning of all parameters is conserved (i.e. that cell density is defined per unit volume). Values and ranges for the necessary material parameters are taken from the literature and from our experimental procedure (Table 1). Note that we use subscript *c* and *s* to differentiate the cortical and subcortical regions, with a Heaviside function $\hat{H}\left(•\right)$ of radial position *r* to describe the the smooth spatial transition between them.

**Neurogenesis**. We assume that the ventricular zone covers a region of 0.2*R*_0_ [25], such that the spatial distribution *G*^*x*^(*r*) is given by

$$\begin{array}{c} G^{x}\left(r\right)=\hat{H}(r-\delta^{x};\alpha_{G}), \end{array}$$ (16)

where *δ*^*x*^ = 0.2*R*_0_ is the location of ventricular boundary, and *α*_*G*_ is a smoothing parameter (Fig 11B). For the temporal distribution, we aim to capture the neurons that have been *in utero* electroporated at different times as done in the experiment. Thus we define $G_{i}^{t}\left(t\right)$ as a delta function, viz.,

$$\begin{array}{c} G_{i}^{t}\left(t\right)=\frac{\epsilon^{2}}{{\left(t-\delta_{i}^{t}\right)}^{2}+\epsilon^{2}}, \end{array}$$ (17)

where *ϵ* controls the smoothness of the function (Fig 11C). Additionally, $\delta_{i}^{t}=\left[0,3,6\right]\;\text{d}$ is the electroporation time for three cohort of neurons.

**Neuron density flux**. As we reported previously [38, 56], neurons in deeper cortical layers 5/6 were born earlier (IUE at E31), neurons found in superficial cortical layers 2/3 were born later (IUE at E37), and neurons born at the mediate time (IUE at E34) were consistently found in the medium cortical layer 4 (Fig 5, circles). Similar observations in ferret brains have been reported by [8], in which they used ^3^H-thymidine to trace cohorts of neurons over time. This positioning is likely guided by a gradient of chemoattractant [65]. To capture this behavior, we define the velocity magnitude in Eq (11) as

$$\begin{array}{c} {\hat{v}}_{i}\left(r\right)=v_{i}\hat{H}\left(\delta_{i}^{v}-r;\alpha_{v}\right)-0.5v_{i}, \end{array}$$ (18)

where *v*_*i*_ is the baseline velocity constant, $\delta_{i}^{v}$ is the final destination of *i*th cohort of neurons, and *α*_*v*_ is a smoothing parameter. It is worth noting that we shift the entire function vertically by 0.5*v*_*i*_ so that cell migration ceases at the location of zero velocities (Fig 11D). Unlike previous models [25, 26], the diffusion coefficient *D* is taken to be constant throughout the brain.

**Growth parameters**. As the literature suggests that the cortex expands more tangentially than radially [11], the profiles of radial and tangential growth parameter are given by

$$\begin{array}{c} \begin{array}{cc} & k^{\|}\left(r\right)=k_{s}+k_{s}\left(1/\beta_{k}-1\right)\hat{H}\left(r-\delta_{k};\alpha_{k}\right)\;\text{and}\\ & k^{⊥}\left(r\right)=k_{s}+k_{s}\left(\beta_{k}-1\right)\hat{H}\left(r-\delta_{k};\alpha_{k}\right), \end{array} \end{array}$$ (19)

where *k*_*s*_ is the growth parameter of subcortex, *δ*_*k*_ is the location of growth mode transition, and *α*_*k*_ is the smoothing parameter (Fig 11E). Initially, we assume tangential-radial growth ratio *β*_*k*_ = 1 [25], but later consider a reasonable range of growth ratio factor *β*_*k*_ = [1, 1.5, 2].

**Mechanical properties**. The profile of shear modulus *μ* in Eq (15) is given by

$$\begin{array}{c} \mu \left(r\right)=\mu_{s}+\mu_{s}\left(\beta_{\mu }-1\right)\hat{H}\left(r-\delta_{\mu };\alpha_{\mu }\right), \end{array}$$ (20)

where *β*_*μ*_ = *μ*_*c*_/*μ*_*s*_ is stiffness ratio between cortex and subcortex, *δ*_*μ*_ is the location of modulus transition, and *α*_*μ*_ is the smoothing parameter (Fig 11F). The shear modulus for the subcortex is *μ*_*s*_ = 1 kPa. The Poisson’s ratio is defined as *ν* = 0.45, such that the Lamé constant for the subcortex is *L*_*s*_ = 9.3 kPa [45]. It has been shown that axon elongation makes subcortical tissue more “compliant” on the timescales of folding [34, 46, 66], thus, we assume stiffness ratio *β*_*μ*_ = 3, but later we consider a reasonable range of stiffness ratio of *β*_*μ*_ = [3, 5, 7, 9].

### 4.5 Numerical model

Our finite-element procedures are implemented in Abaqus/Standard (2020) [40]. We present the details of numerical implementation and the code verification in S1 Appendix.

For model calibration, we take the computational domain as a 3-D slender bar with an initial length/radius of *R*_0_ = 239 μm, consisting of 60 brick elements (U3D8) in Abaqus/Standard (2020) [40]. This was chosen to represent the ROIs analyzed from our experimental data. One end is fully fixed, while the long edges are assigned symmetry boundary conditions such that the solution only varies in 1-D, along the length. The entire bar is free to move along the x-axis, which coincides with the direction of cell velocity, **N** = [1, 0, 0]^⊤^. For simplicity, we do not consider any tangential growth, i.e., *k*^⊥^ = 0 in Eq (13).

In cortical folding simulations, both the plane-strain half circle and one-eighth sphere are discretized into 1147 four-noded quadrilateral elements (UPE4) and 361 eight-noded brick elements (U3D8) in Abaqus. In both domains, the cut faces or edges are assigned symmetry boundary conditions, while the curved exterior faces or edges are traction-free and allowed to self contact. Thus, the plane-strain half circle simplifies to a 2-D solution field, while the partial sphere yields a fully 3-D solution. For biological boundary conditions, zero cell density flux are prescribed at all boundaries. The neuronal migration is radially aligned with the unit vector fields of **N** = [*x*, *y*, 0]^⊤^ and **N** = [*x*, *y*, *z*]^⊤^ for 2-D and 3-D cases, respectively.

### 4.6 Genetic algorithm

The genetic algorithm is used along with Python scripts to generate input files, execute jobs, and access the output database automatically in Abaqus (Table 3). It generates 10 genomes in every generation, each with a corresponding Abaqus input file with the current set of material parameters. After running the simulation, we collect the simulated results from the Abaqus output database, including domain lengths at the three preparation times $\left\{l_{t1}^{\text{sim}},l_{t2}^{\text{sim}},l_{t3}^{\text{sim}}\right\}$ and eight cell density vectors $\left\{c_{1,t1}^{\text{sim}},c_{2,t1}^{\text{sim}},c_{1,t2}^{\text{sim}},c_{2,t2}^{\text{sim}},c_{3,t2}^{\text{sim}},c_{1,t3}^{\text{sim}},c_{2,t3}^{\text{sim}},c_{3,t3}^{\text{sim}}\right\}$ that store the density of each cohort across the domain’s length at each preparation time. We define an objective function that measures normalized errors between the simulations and experiments,

$$\begin{array}{c} \begin{array}{cc} f_{\text{obj}}= & w_{1}(\frac{\|c_{1,t1}^{\text{sim}}-c_{1,t1}^{\text{exp}}\|}{\|c_{1,t1}^{\text{exp}}\|}+\frac{\|c_{2,t1}^{\text{sim}}-c_{2,t1}^{\text{exp}}\|}{\|c_{2,t1}^{\text{exp}}\|}+\frac{\|c_{1,t2}^{\text{sim}}-c_{1,t2}^{\text{exp}}\|}{\|c_{1,t2}^{\text{exp}}\|}+\frac{\|c_{2,t2}^{\text{sim}}-c_{2,t2}^{\text{exp}}\|}{\|c_{2,t2}^{\text{exp}}\|}\\ & +\frac{\|c_{3,t2}^{\text{sim}}-c_{3,t2}^{\text{exp}}\|}{\|c_{3,t2}^{\text{exp}}\|}+\frac{\|c_{1,t3}^{\text{sim}}-c_{1,t3}^{\text{exp}}\|}{\|c_{1,t3}^{\text{exp}}\|}+\frac{\|c_{2,t3}^{\text{sim}}-c_{2,t3}^{\text{exp}}\|}{\|c_{2,t3}^{\text{exp}}\|}+\frac{\|c_{3,t3}^{\text{sim}}-c_{3,t3}^{\text{exp}}\|}{\|c_{3,t3}^{\text{exp}}\|})\\ & +w_{2}(\frac{\|l_{t1}^{\text{sim}}-l_{t1}^{\text{exp}}\|}{l_{t1}^{\text{exp}}}+\frac{\|l_{t2}^{\text{sim}}-l_{t2}^{\text{exp}}\|}{l_{t2}^{\text{exp}}}+\frac{\|l_{t3}^{\text{sim}}-l_{t3}^{\text{exp}}\|}{l_{t3}^{\text{exp}}}) \end{array}, \end{array}$$ (21)

where two weights *w*_1_ = 11/8 and *w*_2_ = 11/3 are used to balance the contributions of density and displacement errors to the objective function.

**Table 3:** Algorithm for the calibration of our model parameters using a genetic algorithm.

generate initial parameter genomes **if** not converged: **for** each genome in the generation generate Abaqus input file execute Abaqus job obtain deformations and cell densities from output database compute objective function *f*_obj_ **end for** check convergence **end if**

## Supporting information

S1 Appendix Details of finite element implementation, code verification, and the robustness of the genetics algorithm. (PDF)

## References

[1] Herculano-Houzel S. The human brain in numbers: a linearly scaled-up primate brain. Frontiers in human neuroscience. 2009;3:31. doi: 10.3389/neuro.09.031.2009 19915731 PMC2776484

[2] Mota B, Herculano-Houzel S. Cortical folding scales universally with surface area and thickness, not number of neurons. Science. 2015;349(6243):74–77. doi: 10.1126/science.aaa9101 26138976

[3] Welker W. Why does cerebral cortex fissure and fold? In: Cerebral cortex. Springer; 1990. p. 3–136.

[4] Nordahl CW, Dierker D, Mostafavi I, Schumann CM, Rivera SM, Amaral DG, et al. Cortical folding abnormalities in autism revealed by surface-based morphometry. Journal of Neuroscience. 2007;27(43):11725–11735. doi: 10.1523/JNEUROSCI.0777-07.2007 17959814 PMC6673212

[5] Jou RJ, Hardan AY, Keshavan MS. Reduced Cortical Folding in Individuals at High Risk for Schizophrenia: A Pilot Study. Schizophrenia Research. 2005;75(2-3):309–313. doi: 10.1016/j.schres.2004.11.008 15885522

[6] Harvey AS, Mandelstam SA, Maixner WJ, Leventer RJ, Semmelroch M, MacGregor D, et al. The Surgically Remediable Syndrome of Epilepsy Associated with Bottom-of-Sulcus Dysplasia. Neurology. 2015;84(20):2021–2028. doi: 10.1212/WNL.0000000000001591 25888556 PMC4442099

[7] Zecevic N, Chen Y, Filipovic R. Contributions of cortical subventricular zone to the development of the human cerebral cortex. Journal of Comparative Neurology. 2005;491(2):109–122. doi: 10.1002/cne.20714 16127688 PMC2628573

[8] Jackson CA, Peduzzi JD, Hickey T. Visual cortex development in the ferret. I. Genesis and migration of visual cortical neurons. Journal of Neuroscience. 1989;9(4):1242–1253. doi: 10.1523/JNEUROSCI.09-04-01242.1989 2703875 PMC6569864

[9] Gilmore EC, Herrup K. Cortical development: layers of complexity. Current Biology. 1997;7(4):R231–R234. doi: 10.1016/S0960-9822(06)00108-4 9162498

[10] Marín O, Valiente M, Ge X, Tsai LH. Guiding neuronal cell migrations. Cold Spring Harbor perspectives in biology. 2010;2(2):a001834. doi: 10.1101/cshperspect.a001834 20182622 PMC2828271

[11] Budday S, Steinmann P III, Kuhl E. Physical biology of human brain development. Frontiers in cellular neuroscience. 2015;9:257. doi: 10.3389/fncel.2015.00257 26217183 PMC4495345

[12] Kroenke CD, Bayly PV. How forces fold the cerebral cortex. Journal of Neuroscience. 2018;38(4):767–775. doi: 10.1523/JNEUROSCI.1105-17.2017 29367287 PMC5783962

[13] Borrell V, Götz M. Role of radial glial cells in cerebral cortex folding. Current opinion in neurobiology. 2014;27:39–46. doi: 10.1016/j.conb.2014.02.007 24632307

[14] Chavoshnejad P, Li X, Zhang S, Dai W, Vasung L, Liu T, et al. Role of Axonal Fibers in the Cortical Folding Patterns: A Tale of Variability and Regularity. Brain Multiphysics. 2021; p. 100029. doi: 10.1016/j.brain.2021.100029

[15] Borrell V. How cells fold the cerebral cortex. Journal of Neuroscience. 2018;38(4):776–783. doi: 10.1523/JNEUROSCI.1106-17.2017 29367288 PMC6596235

[16] Barron DH. An experimental analysis of some factors involved in the development of the fissure pattern of the cerebral cortex. Journal of Experimental Zoology. 1950;113(3):553–581. doi: 10.1002/jez.1401130304

[17] Richman DP, Stewart RM, Hutchinson JW, Caviness VS. Mechanical model of brain convolutional development. Science. 1975;189(4196):18–21. doi: 10.1126/science.1135626 1135626

[18] Tallinen T, Chung JY, Biggins JS, Mahadevan L. Gyrification from constrained cortical expansion. Proceedings of the National Academy of Sciences. 2014;111(35):12667–12672. doi: 10.1073/pnas.1406015111 25136099 PMC4156754

[19] Van Essen DC. A tension-based theory of morphogenesis and compact wiring in the central nervous system. Nature. 1997;385(6614):313–318. doi: 10.1038/385313a0 9002514

[20] Xu G, Knutsen AK, Dikranian K, Kroenke CD, Bayly PV, Taber LA. Axons Pull on the Brain, But Tension Does Not Drive Cortical Folding. Journal of Biomechanical Engineering. 2010;132(7). doi: 10.1115/1.4001683 PMC3170872 20590291

[21] Striegel DA, Hurdal MK. Chemically based mathematical model for development of cerebral cortical folding patterns. PLoS computational biology. 2009;5(9):e1000524. doi: 10.1371/journal.pcbi.1000524 19779554 PMC2740831

[22] Lefèvre J, Mangin JF. A reaction-diffusion model of human brain development. PLoS computational biology. 2010;6(4):e1000749. doi: 10.1371/journal.pcbi.1000749 20421989 PMC2858670

[23] Darayi M, Hoffman ME, Sayut J, Wang S, Demirci N, Consolini J, et al. Computational models of cortical folding: A review of common approaches. Journal of Biomechanics. 2021; p. 110851. doi: 10.1016/j.jbiomech.2021.110851 34802706

[24] Verner S, Garikipati K. A computational study of the mechanisms of growth-driven folding patterns on shells, with application to the developing brain. Extreme Mechanics Letters. 2018;18:58–69. doi: 10.1016/j.eml.2017.11.003

[25] de Rooij R, Kuhl E. A physical multifield model predicts the development of volume and structure in the human brain. Journal of the Mechanics and Physics of Solids. 2018;112:563–576. doi: 10.1016/j.jmps.2017.12.011

[26] Zarzor M, Kaessmair S, Steinmann P, Blümcke I, Budday S. A two-field computational model couples cellular brain development with cortical folding. Brain Multiphysics. 2021;2:100025. doi: 10.1016/j.brain.2021.100025

[27] Takeda H, Kameo Y, Adachi T. Continuum modeling for neuronal lamination during cerebral morphogenesis considering cell migration and tissue growth. Computer Methods in Biomechanics and Biomedical Engineering. 2021;24(7):799–805. doi: 10.1080/10255842.2020.1852554 33290089

[28] Smart I, McSherry G. Gyrus formation in the cerebral cortex in the ferret. I. Description of the external changes. Journal of anatomy. 1986;146:141. 3693054 PMC1166530

[29] Smart I, McSherry G. Gyrus formation in the cerebral cortex of the ferret. II. Description of the internal histological changes. Journal of anatomy. 1986;147:27. 3693076 PMC1261544

[30] Bayly P, Taber L, Kroenke C. Mechanical forces in cerebral cortical folding: a review of measurements and models. Journal of the mechanical behavior of biomedical materials. 2014;29:568–581. doi: 10.1016/j.jmbbm.2013.02.018 23566768 PMC3842388

[31] Neal J, Takahashi M, Silva M, Tiao G, Walsh CA, Sheen VL. Insights into the gyrification of developing ferret brain by magnetic resonance imaging. Journal of anatomy. 2007;210(1):66–77. doi: 10.1111/j.1469-7580.2006.00674.x 17229284 PMC2100265

[32] Barnette AR, Neil JJ, Kroenke CD, Griffith JL, Epstein AA, Bayly PV, et al. Characterization of brain development in the ferret via MRI. Pediatric research. 2009;66(1):80–84. doi: 10.1203/PDR.0b013e3181a291d9 19287340 PMC3384539

[33] Budday S, Nay R, de Rooij R, Steinmann P, Wyrobek T, Ovaert TC, et al. Mechanical properties of gray and white matter brain tissue by indentation. Journal of the mechanical behavior of biomedical materials. 2015;46:318–330. doi: 10.1016/j.jmbbm.2015.02.024 25819199 PMC4395547

[34] Reillo I, Borrell V. Germinal zones in the developing cerebral cortex of ferret: ontogeny, cell cycle kinetics, and diversity of progenitors. Cerebral cortex. 2012;22(9):2039–2054. doi: 10.1093/cercor/bhr284 21988826

[35] Chan A, Chong K, Martinovich C, Simerly C, Schatten G. Transgenic monkeys produced by retroviral gene transfer into mature oocytes. Science. 2001;291(5502):309–312. doi: 10.1126/science.291.5502.309 11209082

[36] Lois C, Hong EJ, Pease S, Brown EJ, Baltimore D. Germline transmission and tissue-specific expression of transgenes delivered by lentiviral vectors. Science. 2002;295(5556):868–872. doi: 10.1126/science.1067081 11786607

[37] Sasaki E, Suemizu H, Shimada A, Hanazawa K, Oiwa R, Kamioka M, et al. Generation of transgenic non-human primates with germline transmission. Nature. 2009;459(7246):523–527. doi: 10.1038/nature08090 19478777

[38] Kawasaki H, Iwai L, Tanno K. Rapid and efficient genetic manipulation of gyrencephalic carnivores using in utero electroporation. Molecular Brain. 2012;5(1):1–7. doi: 10.1186/1756-6606-5-24 22716093 PMC3460770

[39] Kawasaki H. Molecular investigations of development and diseases of the brain of higher mammals using the ferret. Proceedings of the Japan Academy, Series B. 2017;93(5):259–269. doi: 10.2183/pjab.93.017 28496051 PMC5489433

[40] Abaqus/Standard. Abaqus Reference Manuals. Providence, RI: Dassault Systemes Simulia; 2020.

[41] Kawasaki H, Toda T, Tanno K. In vivo genetic manipulation of cortical progenitors in gyrencephalic carnivores using in utero electroporation. Biology Open. 2013;2(1):95–100. doi: 10.1242/bio.20123160 23336081 PMC3545273

[42] Kawasaki H, Mizuseki K, Nishikawa S, Kaneko S, Kuwana Y, Nakanishi S, et al. Induction of midbrain dopaminergic neurons from ES cells by stromal cell–derived inducing activity. neuron. 2000;28(1):31–40. doi: 10.1016/S0896-6273(00)00083-0 11086981

[43] Toda T, Homma D, Tokuoka H, Hayakawa I, Sugimoto Y, Ichinose H, et al. Birth regulates the initiation of sensory map formation through serotonin signaling. Developmental cell. 2013;27(1):32–46. doi: 10.1016/j.devcel.2013.09.002 24135230

[44] Schneider CA, Rasband WS, Eliceiri KW. NIH Image to ImageJ: 25 years of image analysis. Nature methods. 2012;9(7):671–675. doi: 10.1038/nmeth.2089 22930834 PMC5554542

[45] Iwashita M, Nomura T, Suetsugu T, Matsuzaki F, Kojima S, Kosodo Y. Comparative analysis of brain stiffness among amniotes using glyoxal fixation and atomic force microscopy. Frontiers in cell and developmental biology. 2020;8:938. doi: 10.3389/fcell.2020.574619 33043008 PMC7517470

[46] Holland MA, Miller KE, Kuhl E. Emerging brain morphologies from axonal elongation. Annals of biomedical engineering. 2015;43(7):1640–1653. doi: 10.1007/s10439-015-1312-9 25824370 PMC4497873

[47] Nichols AJ, Carney LH, Olson EC. Comparison of slow and fast neocortical neuron migration using a new in vitro model. BMC neuroscience. 2008;9(1):1–13. doi: 10.1186/1471-2202-9-50 18534012 PMC2440755

[48] Burlakov V, Taylor R, Koerner J, Emptage N. Analysis of microscopic parameters of single-particle trajectories in neurons. Biophysical journal. 2010;99(5):1368–1376. doi: 10.1016/j.bpj.2010.06.021 20816048 PMC2931737

[49] Wright AH. Genetic algorithms for real parameter optimization. In: Foundations of genetic algorithms. vol. 1. Elsevier; 1991. p. 205–218.

[50] Holland M, Budday S, Goriely A, Kuhl E. Symmetry breaking in wrinkling patterns: Gyri are universally thicker than sulci. Physical review letters. 2018;121(22):228002. doi: 10.1103/PhysRevLett.121.228002 30547630

[51] Budday S, Steinmann P. On the influence of inhomogeneous stiffness and growth on mechanical instabilities in the developing brain. International Journal of Solids and Structures. 2018;132:31–41. doi: 10.1016/j.ijsolstr.2017.08.010

[52] Wang S, Demirci N, Holland MA. Numerical investigation of biomechanically coupled growth in cortical folding. Biomechanics and Modeling in Mechanobiology. 2021;20(2):555–567. doi: 10.1007/s10237-020-01400-w 33151429

[53] Hamabe-Horiike T, Kawasaki K, Sakashita M, Ishizu C, Yoshizaki T, Harada Si, et al. Glial cell type-specific gene expression in the mouse cerebrum using the piggyBac system and in utero electroporation. Scientific reports. 2021;11(1):1–14. doi: 10.1038/s41598-021-84210-z 33649472 PMC7921133

[54] Pfister BJ, Iwata A, Meaney DF, Smith DH. Extreme stretch growth of integrated axons. Journal of Neuroscience. 2004;24(36):7978–7983. doi: 10.1523/JNEUROSCI.1974-04.2004 15356212 PMC6729931

[55] Garcia-Gonzalez D, Muñoz-Barrutia A. Computational insights into the influence of substrate stiffness on collective cell migration. Extreme Mechanics Letters. 2020;40:100928. doi: 10.1016/j.eml.2020.100928

[56] Shinmyo Y, Terashita Y, Duong TAD, Horiike T, Kawasumi M, Hosomichi K, et al. Folding of the cerebral cortex requires Cdk5 in upper-layer neurons in gyrencephalic mammals. Cell Reports. 2017;20(9):2131–2143. doi: 10.1016/j.celrep.2017.08.024 28854363

[57] Matsumoto N, Shinmyo Y, Ichikawa Y, Kawasaki H. Gyrification of the cerebral cortex requires FGF signaling in the mammalian brain. Elife. 2017;6:e29285. doi: 10.7554/eLife.29285 29132503 PMC5685484

[58] Matsumoto N, Tanaka S, Horiike T, Shinmyo Y, Kawasaki H. A discrete subtype of neural progenitor crucial for cortical folding in the gyrencephalic mammalian brain. Elife. 2020;9:e54873. doi: 10.7554/eLife.54873 32312384 PMC7173966

[59] Kawasaki H, Crowley JC, Livesey FJ, Katz LC. Molecular organization of the ferret visual thalamus. Journal of Neuroscience. 2004;24(44):9962–9970. doi: 10.1523/JNEUROSCI.2165-04.2004 15525781 PMC6730254

[60] Iwai L, Kawasaki H. Molecular development of the lateral geniculate nucleus in the absence of retinal waves during the time of retinal axon eye-specific segregation. Neuroscience. 2009;159(4):1326–1337. doi: 10.1016/j.neuroscience.2009.02.010 19409202

[61] Sehara K, Toda T, Iwai L, Wakimoto M, Tanno K, Matsubayashi Y, et al. Whisker-related axonal patterns and plasticity of layer 2/3 neurons in the mouse barrel cortex. Journal of Neuroscience. 2010;30(8):3082–3092. doi: 10.1523/JNEUROSCI.6096-09.2010 20181605 PMC6633930

[62] Rodriguez EK, Hoger A, McCulloch AD. Stress-dependent finite growth in soft elastic tissues. Journal of biomechanics. 1994;27(4):455–467. doi: 10.1016/0021-9290(94)90021-3 8188726

[63] Rakic P. Specification of cerebral cortical areas. Science. 1988;241(4862):170–176. doi: 10.1126/science.3291116 3291116

[64] Rakic P. Principles of neural cell migration. Experientia. 1990;46(9):882–891. doi: 10.1007/BF01939380 2209797

[65] Aman A, Piotrowski T. Cell migration during morphogenesis. Developmental biology. 2010;341(1):20–33. doi: 10.1016/j.ydbio.2009.11.014 19914236

[66] Garcia KE, Wang X, Kroenke CD. A model of tension-induced fiber growth predicts white matter organization during brain folding. Nature communications. 2021;12(1):1–13. doi: 10.1038/s41467-021-26971-9 PMC8602459 34795256

