# Supplementary material for: Orchestrated neuronal migration and cortical folding: A computational and experimental study
Source: PLoS Comput Biol. 2022 Jun 16;18(6):e1010190. doi: 10.1371/journal.pcbi.1010190 (PMC9258886; doi:10.1371/journal.pcbi.1010190)
Supplement: S1 Appendix — (PDF) [file pcbi.1010190.s001.pdf]

S1 Appendix for: Orchestrated neuronal migration and cortical folding: A computational and experimental study

Shuolun Wang, Kengo Saito, Hiroshi Kawasaki, Maria A. Holland

May 17, 2022

Finite element implementation

First, we briefly summarize the strong form of the governing equations in Section 4.4. The strong form of the coupled PDEs are given by

$$\begin{cases} \left.\begin{aligned} \dot{c}_i + c_i\frac{\dot{J}}{J} - \operatorname{div}\mathbf{q}_i - f_i^c = 0 \quad &\text{in} \quad \mathcal{B}_t, \\ c_i = \breve{c}_i \quad &\text{on} \quad \mathcal{S}_c, \end{aligned}\right\} \text{Balance of cell density} \\ \left.\begin{aligned} \operatorname{div}\mathbf{T} = \mathbf{0} \quad &\text{in} \quad \mathcal{B}_t, \\ \mathbf{u} = \breve{\mathbf{u}} \quad &\text{on} \quad \mathcal{S}_{\mathbf{u}}, \\ \mathbf{T}\mathbf{n} = \breve{\mathbf{t}} \quad &\text{on} \quad \mathcal{S}_{\mathbf{t}}. \end{aligned}\right\} \text{Balance of momentum} \end{cases} \tag{S.1}$$

Next, we let w_1, $\mathbf{w}_2$ denote two test fields which vanish on $\mathcal{S}_c$ and $\mathcal{S}_{\mathbf{u}}$, respectively. After multiplying the PDEs with two test fields and performing integration by parts, the weak form could be expressed as

$$\begin{cases} \displaystyle\int_{\mathcal{B}_t} w_1\Big(\dot{c}_i + c_i\frac{\dot{J}}{J}\Big)dv + \int_{\mathcal{B}_t}\big(\operatorname{grad} w_1 \cdot \mathbf{q}_i\big)dv - \int_{\mathcal{B}_t}\big(w_1 f_i^c\big)dv = 0, \\ \displaystyle\int_{\mathcal{B}_t}\big(\mathbf{T} : \operatorname{grad}\mathbf{w}_2\big)dv = \int_{\mathcal{S}_{\mathbf{t}}}\big(\mathbf{w}_2 \cdot \breve{\mathbf{t}}\big)da. \end{cases} \tag{S.2}$$

As is routine, the body is discretized into finite elements such that $\mathcal{B}_t = \cup\mathcal{B}_t^e$, and the nodal variables are taken to be the cell densities and displacement, which are interpolated inside each element by

$$c_i = \sum c_i^A N^A \qquad \text{and} \quad \mathbf{u} = \sum \mathbf{u}^A N^A. \tag{S.3}$$

with the index $A = 1, 2, ...$ denoting the nodes of the element, c_i^A and $\mathbf{u}^A$ are the nodal cell densities and displacements, respectively, and N^A the shape functions. Next, the test fields w_1 and $\mathbf{w}_2$ are interpolated by the same shape functions in the Galerkin approach, viz.

$$w_1 = \sum w_1^A N^A, \quad \text{and} \quad \mathbf{w}_2 = \sum \mathbf{w}_2^A N^A. \tag{S.4}$$

After substitution of Eq (S.3) and Eq (S.4) into the weak forms in Eq (S.2), we arrive at element-level of residuals. Since the expressions are similar for different neuron types, we only demonstrate the residual for $c_1(\mathbf{x}, t)$,

$$R_{c_1}^A = \int_{\mathcal{B}_t^e} N^A\left(\dot{c}_1 + c_1\frac{\dot{J}}{J}\right) + \left(\operatorname{grad} N^A \cdot \mathbf{q}_1\right)dv - \left(N^A f_1^c\right)dv\,. \tag{S.5}$$

The element level residual for the displacement field $\mathbf{u}(\mathbf{x}, t)$ is given by

$$\mathbf{R}_{\mathbf{u}}^A = -\int_{\mathcal{B}_t^e}\left(\mathbf{T}\cdot\operatorname{grad} N^A\right)dv + \int_{\mathcal{S}_{\mathbf{t}}^e}(N^A\breve{\mathbf{t}})da. \tag{S.6}$$

Since we consider cohorts of electroporated neurons at three different times in this work, four element-level residuals in total are assembled into a global residual, which, when set to zero, represents a system of non-linear equations for the nodal degrees of freedom.

The following tangents

$$(\mathbf{K}_{\mathbf{uu}})^{AB} = -\frac{\partial R_{\mathbf{u}}^A}{\partial \mathbf{u}^{\mathbf{B}}},\quad (\mathbf{K}_{\mathbf{u}c_1})^{AB} = -\frac{\partial R_{\mathbf{u}}^A}{\partial c_1^B},\quad (\mathbf{K}_{\mathbf{u}c_2})^{AB} = -\frac{\partial R_{\mathbf{u}}^A}{\partial c_2^B},\quad (\mathbf{K}_{\mathbf{u}c_3})^{AB} = -\frac{\partial R_{\mathbf{u}}^A}{\partial c_3^B},$$

$$(\mathbf{K}_{c_1\mathbf{u}})^{AB} = -\frac{\partial R_{c_1}^A}{\partial \mathbf{u}^B},\quad (K_{c_1c_1})^{AB} = -\frac{\partial R_{c_1}^A}{\partial c_1^B},\quad (K_{c_1c_2})^{AB} = -\frac{\partial R_{c_1}^A}{\partial c_2^B},\quad (K_{c_1c_3})^{AB} = -\frac{\partial R_{c_1}^A}{\partial c_3^B},$$

$$(\mathbf{K}_{c_2\mathbf{u}})^{AB} = -\frac{\partial R_{c_2}^A}{\partial \mathbf{u}^B},\quad (K_{c_2c_1})^{AB} = -\frac{\partial R_{c_2}^A}{\partial c_1^B},\quad (K_{c_2c_2})^{AB} = -\frac{\partial R_{c_2}^A}{\partial c_2^B},\quad (K_{c_2c_3})^{AB} = -\frac{\partial R_{c_2}^A}{\partial c_3^B},$$

$$(\mathbf{K}_{c_3\mathbf{u}})^{AB} = -\frac{\partial R_{c_3}^A}{\partial \mathbf{u}^B},\quad (K_{c_3c_1})^{AB} = -\frac{\partial R_{c_3}^A}{\partial c_1^B},\quad (K_{c_3c_2})^{AB} = -\frac{\partial R_{c_3}^A}{\partial c_2^B},\quad (K_{c_3c_3})^{AB} = -\frac{\partial R_{c_3}^A}{\partial c_3^B},$$

are required by the iterative Newton-Raphson procedure for convergence.

More specifically, the tangent $K_{\mathbf{uu}}^{AB}$ is given by

$$K_{u_iu_k}^{AB} = -\int_{\mathcal{B}_t^e}\frac{\partial N^A}{\partial x_j}\mathbb{A}_{ijkl}\frac{\partial N^B}{\partial x_l}dv - \int_{\mathcal{S}_{\mathbf{t}}^e} N^A N^B\frac{\partial \breve{t}_i}{\partial u_k}da, \tag{S.7}$$

where the spatial tangent modulus $\mathbb{A}$ is related to referential tangent modulus $\mathbb{A}_{\mathrm{R}}$ through

$$\mathbb{A}_{ijkl} = J^{-1}F_{jm}F_{ln}(\mathbb{A}_{\mathrm{R}})_{imkn}, \tag{S.8}$$

and the referential tangent modulus is given by $\mathbb{A}_{\mathrm{R}} \overset{\text{def}}{=} \dfrac{\partial \mathbf{T}_{\mathrm{R}}}{\partial \mathbf{F}}$ with $\mathbf{T}_{\mathrm{R}} = J\mathbf{T}\mathbf{F}^{-\top}$ denoted as the Piola stress [1]. After using Eq (15), the Piola stress is now given by

$$\mathbf{T}_{\mathrm{R}} = J^{\mathrm{g}}\left([\lambda\ln J^{\mathrm{e}} - \mu]\mathbf{F}^{-\top} + \mu\mathbf{F}\mathbf{F}^{\mathrm{g}-1}\mathbf{F}^{\mathrm{g}-\top}\right), \tag{S.9}$$

and hence

$$\begin{aligned}(\mathbb{A}_{\mathrm{R}})_{ijkl} =& J^{\mathrm{g}}\left([\lambda\ln J^{\mathrm{e}} - \mu + \lambda]F_{ji}^{-1}F_{lk}^{-1} + \mu F_{ia}F_{lk}^{-1}F_{ab}^{\mathrm{g}-1}F_{jb}^{\mathrm{g}-1}\right)\\ &+ J^{\mathrm{g}}\left([\mu - \lambda\ln J^{\mathrm{e}}]F_{li}^{-1}F_{jk}^{-1} + \mu\delta_{ik}F_{lb}^{\mathrm{g}-1}F_{jb}^{\mathrm{g}-1}\right).\end{aligned} \tag{S.10}$$

The three diagonal tangents for neuron density are similar, and here we only demonstrate $K^{AB}_{c_1c_1}$, which is given by

$$\begin{aligned}
K^{AB}_{c_1c_1} &= -\int_{\mathcal{B}^e_t} N^A N^B \left(\frac{1}{\Delta t} + \frac{\dot{J}}{J}\right) dv - \int_{\mathcal{B}^e_t} \operatorname{grad} N^A \cdot \left(\frac{\partial \mathbf{q}_1}{\partial c_1}\right) N^B dv \\
&= -\int_{\mathcal{B}^e_t} N^A N^B \left(\frac{1}{\Delta t} + \frac{\dot{J}}{J}\right) dv \\
&\quad - \int_{\mathcal{B}^e_t} \operatorname{grad} N^A \cdot \mathbf{D} \cdot \operatorname{grad} N^B dv \\
&\quad - \int_{\mathcal{B}^e_t} \operatorname{grad} N^A \left(\breve{\mathcal{H}} + c_1 \frac{\partial \breve{\mathcal{H}}}{\partial c_1}\right) \cdot \mathbf{v} N^B dv,
\end{aligned} \tag{S.11}$$

where the derivative $\frac{\partial \breve{\mathcal{H}}}{\partial c_1}$ is given by

$$\frac{\partial \breve{\mathcal{H}}}{\partial c_1} = \frac{\alpha_c e^{\alpha_c(c_1-c_0)}}{e^{\alpha_c(c_1-c_0)}+1} - \frac{\alpha_c e^{2\alpha_c(c_1-c_0)}}{(e^{\alpha_c(c_1-c_0)}+1)^2}. \tag{S.12}$$

We note the current work does not address coupling between cohorts of neurons, thus we set $K_{c_1c_2}$, $K_{c_1c_3}$, $K_{c_2c_1}$, $K_{c_2c_3}$, $K_{c_3c_1}$, and $K_{c_3c_2}$ equal to zero.

The displacement–cell density tangents are similar among the three types of neurons; we only demonstrate $\mathbf{K}^{AB}_{u_ic_1}$, which is given by

$$\mathbf{K}^{AB}_{u_ic_1} = -\int_{\mathcal{B}^e_t} \frac{\partial N^A}{\partial x_j} \left(\frac{\partial T_{ij}}{\partial c_1}\right) N^B dv, \tag{S.13}$$

where the derivative $\left(\frac{\partial T}{\partial c_1}\right)_{ij}$ could be further expressed as

$$\left(\frac{\partial T}{\partial c_1}\right)_{ij} = \left(\frac{\partial T}{\partial F^{\mathrm{g}}}\right)_{ijkl} \left(\frac{\partial F^{\mathrm{g}}}{\partial c_1}\right)_{kl}. \tag{S.14}$$

The derivative $\left(\frac{\partial T}{\partial F^{\mathrm{g}}}\right)_{ijkl}$ in Eq (S.14) is given by

$$\begin{aligned}
\left(\frac{\partial T}{\partial F^{\mathrm{g}}}\right)_{ijkl} &= J^{\mathrm{e}-1}\big([\lambda \ln J^{\mathrm{e}} - \mu - \lambda]\delta_{ij} F^{\mathrm{g}-1}_{lk} + \mu B^{\mathrm{e}}_{ij} F^{\mathrm{g}-1}_{kl} \\
&\quad - \mu F^{\mathrm{e}}_{ik} F^{\mathrm{e}}_{ja} F^{\mathrm{g}-1}_{la} - \mu F^{\mathrm{e}}_{ia} F^{\mathrm{e}}_{jk} F^{\mathrm{g}-1}_{la}\big).
\end{aligned} \tag{S.15}$$

The derivative $\left(\frac{\partial F^{\mathrm{g}}}{\partial c_1}\right)_{\mathrm{kl}}$ in Eq (S.14) is given by

$$\left(\frac{\partial F^{\mathrm{g}}}{\partial c_1}\right)_{kl} = k^{\perp}\delta_{kl} + (k^{\parallel} - k^{\perp}) N_k N_l. \tag{S.16}$$

The cell density–displacement tangents among the three neuron types are similar, and we only

demonstrate $\mathbf{K}^{AB}_{c_1 u_k}$, which is given by

$$\begin{aligned}\mathbf{K}^{AB}_{c_1 u_k} = &-\int_{\mathcal{B}^e_t} N^A \big(\dot{c}_1 + c_1/\Delta t + f^c_1\big) dv \\ &+ \int_{\mathcal{B}^e_t} \frac{\partial N^A}{\partial x_k} q_i \frac{\partial N^B}{\partial x_i} dv \\ &- \int_{\mathcal{B}^e_t} \frac{\partial N^A}{\partial x_i} q_i \frac{\partial N^B}{\partial x_k} dv \\ &- \int_{\mathcal{B}^e_t} \frac{\partial N^A}{\partial x_i} \left(\frac{\partial q_i}{\partial F_{kn}} F_{nm} \right)_{ikm} \frac{\partial N^B}{\partial x_m} dv.\end{aligned} \tag{S.17}$$

After implementing a two-noded linear element in Matlab for verification of our numerical implementation (see below), we then implement four-noded quadrilateral and eight-noded brick elements, (UPE4 and U3D8, respectively) in [2] by writing user-defined element (UEL) subroutines, following our previous work [1, 3, 4, 5, 6].

Verification of our numerical implementation

In order to verify our finite element implementations, we decouple the problem and consider each phenomenon (spatiotemporal cell density and mechanical deformation) separately. For the former, we compare our independent numerical solutions in Matlab and Abaqus to each other; in the latter case, we compare our numerical solutions in Abaqus with an analytically tractable solution.

First, we consider cell migration in a rigid slender body with a length of $l = 1\,\mathrm{m}$. Here, we only restrict our attention on one cohort of cells, c_1, and allow them to migrate along x direction. Under these assumptions, the PDE in Eq (5) is now reduced to 1-D,

$$\dot{c}_1 - \frac{dq_1}{dx} - f^c_1 = 0 \quad \text{in} \quad 0 < x \leq l. \tag{S.18}$$

The cell density flux q_1 in Eq (S.18) is given by

$$q_1 = -c_1 \hat{\mathcal{H}}(c_1 - c_0; \alpha_c) \hat{v}_1(x) + D \frac{dc_1}{dx}, \tag{S.19}$$

where the velocity field $\hat{v}_1(x)$ is expressed as

$$\hat{v}_1(x) = v_1 \hat{\mathcal{H}}(\delta^v_1 - x; \alpha_v) - 0.5 v_1. \tag{S.20}$$

The source term f^c_1 in Eq (S.18) is given by the following function,

$$f^c_1 = G^c \left[\frac{\epsilon^2}{(t - \delta^t_1)^2 + \epsilon^2} \right] \hat{\mathcal{H}}(x - \delta^x; \alpha_G). \tag{S.21}$$

For initial and boundary conditions, cell density is absent throughout the domain at $t = 0$, and we assign zero cell flux at the boundary. We consider a 1-D linear element in Matlab and a 3-D eight-noded brick element (U3D8) in Abaqus. The material parameters used for the verification are: $c_0 = 100\,\mathrm{m}^{-3}$, $v_1 = 0.01\,\mathrm{m\,s}^{-1}$, $\alpha_c = 0.1$, $\alpha_G = 100$, $\alpha_v = 100$, $D = 0.005\,\mathrm{m}^2\,\mathrm{s}^{-1}$, $G_c = 25\,\mathrm{m}^{-3}\,\mathrm{s}^{-1}$, $\epsilon = 20\,\mathrm{s}$, $\delta^v_1 = 0.8\,\mathrm{m}$, $\delta^t_1 = 200\,\mathrm{s}$, and $\delta^x = 0.2\,\mathrm{m}$. As expected, both programs produce identical results at $t = 500\,\mathrm{s}$, and thus we consider them verified (SI Fig 1a).

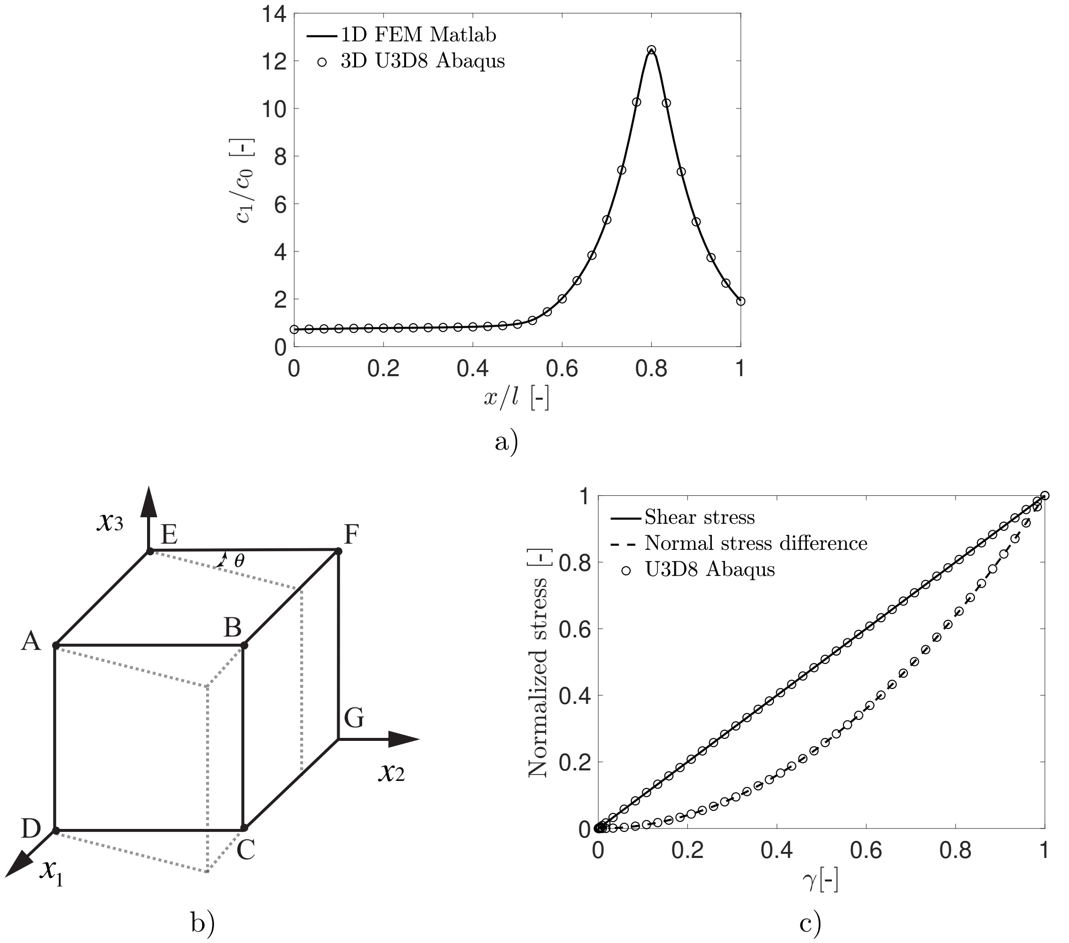

SI Fig 1: Verification of numerical implementations. a) Comparison of numerical solutions of rigid cell migration problem between Matlab and Abaqus; note that the normalized cell density c_1/c_0 is plotted against the normalized length x/l. b) Schematic of a unit cubic undergoing simple shear motion with an angle of θ. c) The normalized shear stress T_{12}/μ and normalized normal stress difference $(T_{11} - T_{33})/\mu$ are plotted against the amount of shear $\gamma = \tan\theta$.

For the mechanical problem, we prescribe a simple shear motion with an angle of θ to a unit cube (SI Fig 1b), such that the associated deformation gradient is given by [7]

$$[\mathbf{F}] = \begin{bmatrix} 1 & \gamma & 0 \\ 0 & 1 & 0 \\ 0 & 0 & 1 \end{bmatrix}, \quad [\mathbf{B}] = \begin{bmatrix} 1+\gamma^2 & \gamma & 0 \\ \gamma & 1 & 0 \\ 0 & 0 & 1 \end{bmatrix}, \quad [\mathbf{C}] = \begin{bmatrix} 1 & \gamma & 0 \\ \gamma & \gamma^2+1 & 0 \\ 0 & 0 & 1 \end{bmatrix}, \tag{S.22}$$

where $\gamma = \tan\theta$ denotes the amount of shear. Next, two assumptions are made make the analytical solution tractable: 1) perfect incompressibility (i.e. $J^{\mathrm{e}} = 1$), and 2) no growth (i.e., $\mathbf{F}^{\mathrm{g}} = \mathbf{1}$). Under these assumptions, the Cauchy stress in Eq (15) is now given by

$$\mathbf{T} = \mu\mathbf{B} + P\mathbf{1}, \tag{S.23}$$

where P is the constitutively indeterminate pressure that is introduced to satisfy the incompressibility constraint. In our simulation, we prescribe the same deformation to a single U3D8 element in Abaqus. Note that in our numerical treatment, we take $L = 10^3\mu$ to approximate a *nearly* incompressible material in the simulation. We compare the analytical solutions for shear stress and normal stress difference, given by

$$T_{12} = \mu\gamma \quad \text{and} \quad T_{11} - T_{33} = \mu\gamma^2, \tag{S.24}$$

respectively, against the numerical solutions (SI Fig 1c). Once again, the excellent agreement between analytical and numerical results indicate the mechanical portion of our finite element implementation is verified.

Robustness of genetic algorithm

To investigate the robustness of the genetic algorithm used in our model calibration, we focus on exploring the evolution of some of the key material parameters. More specifically, we keep track of four key material parameters (G^c, v_i, D, and k_s) and plot them as a function of genome up to 40 generations (SI Fig 2). We note that, under different ranges of initial guesses used in the calibration, most parameters can converge to steady values after roughly about 10 generations/100 genomes. Parameter G^c appears to fall into a local minimum around 100 genomes, before converging to a lower value after approximately 300 genomes. This study reveals the robustness of the genetic algorithm.

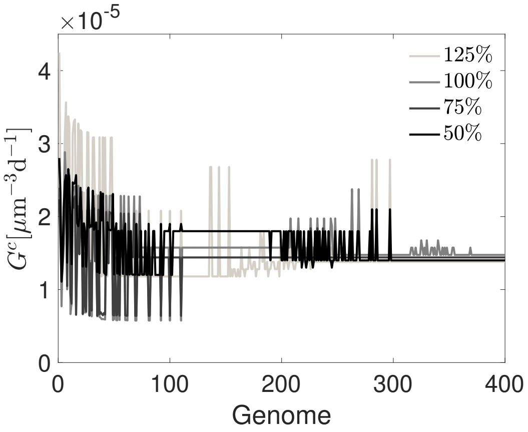

a)

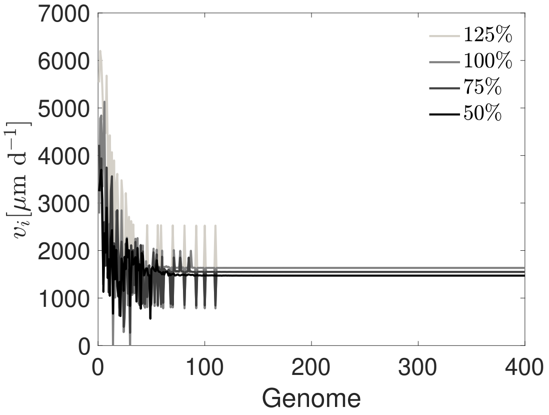

b)

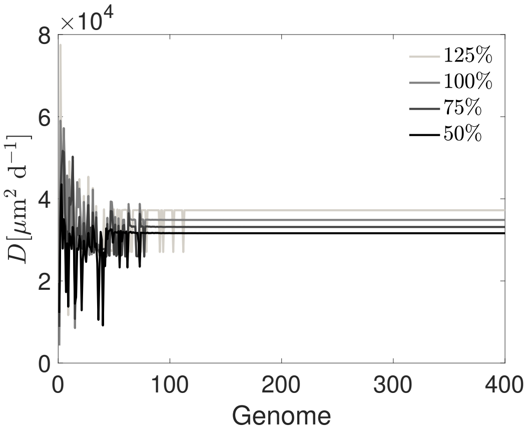

c)

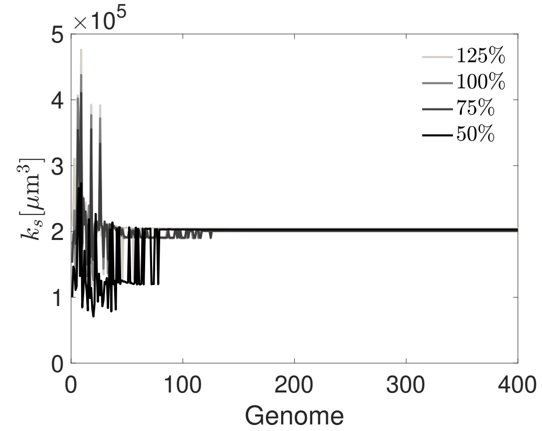

d)

SI Fig 2: Evolution of material parameters of a) baseline division rate G^c, b) migration velocity v_i, c) diffusion coefficient D, and d) subcortical growth parameter k_s as a function of the genome for different ranges of initial guesses.

References

[1] Shawn A. Chester, Claudio V. Di Leo, and Lallit Anand. A finite element implementation of a coupled diffusion-deformation theory for elastomeric gels. *International Journal of Solids and Structures*, 52:1 – 18, 2015.

[2] Abaqus/Standard. *Abaqus Reference Manuals.* Dassault Systemes Simulia, Providence, RI, 2020.

[3] Shuolun Wang, Martina Decker, David L Henann, and Shawn A Chester. Modeling of dielectric viscoelastomers with application to electromechanical instabilities. *Journal of the Mechanics and Physics of Solids*, 95:213–229, 2016.

[4] Craig M Hamel, Fangda Cui, and Shawn A Chester. A finite element method for light activated shape-memory polymers. *International Journal for Numerical Methods in Engineering*, 111(5): 447–473, 2017.

[5] Nikola Bosnjak, Shuolun Wang, Daehoon Han, Howon Lee, and Shawn A Chester. Modeling of fiber-reinforced polymeric gels. *Mechanics Research Communications*, 96:7–18, 2019.

[6] Shuolun Wang and Shawn A Chester. Multi-physics modeling and finite element formulation of corneal uv cross-linking. *Biomechanics and Modeling in Mechanobiology*, pages 1–18, 2021.

[7] Morton E Gurtin, Eliot Fried, and Lallit Anand. *The mechanics and thermodynamics of continua.* Cambridge University Press, 2010.
